# Three‐Dimensional Magnetic Multi‐Metallic Nanozymes With Colorimetric and Catalytic Amplification for Rapid and Highly‐Sensitive Detection of Influenza A

**DOI:** 10.1002/EXP.20240425

**Published:** 2026-05-20

**Authors:** Zhenzhen Liu, Pengyou Zhou, Xiaofei Jia, Xiaoxian Liu, Yong Yang, Yansong Sun, Rui Xiao

**Affiliations:** ^1^ State Key Laboratory of Pathogen and Biosecurity Academy of Military Medical Sciences Beijing China; ^2^ State Key Laboratory of High‐Performance Ceramics and Superfine Microstructures Shanghai Institute of Ceramics Chinese Academy of Sciences Shanghai China

**Keywords:** 3D magnetic multi‐metallic nanozymes, catalytic amplification, influenza A viruses, localized surface plasmon resonance, trifunctional LFIA strips

## Abstract

Lateral flow immunochromatographic assay (LFIA) has been widely used in the point‐of‐care testing field with fast results readout and portability. Nanozymes‐based LFIA strengthened the colorimetric signals of LFIA strips by catalytic oxidation of the colorless substrates into colored substrates, without additional measuring equipment. But the limited specific surface area and functionalized sites of the zero‐dimensional and two‐dimensional nanozymes restricted their peroxidase‐like activity, and the detection sensitivity cannot meet the demand for early diagnosis. Herein, a novel three‐dimensional (3D) magnetic multi‐metallic nanozyme with excellent superparamagnetism and peroxidase‐like activity was developed as a catalytic amplification sensor for the detection of Influenza A (Flu A) viruses. Notably, two‐dimensional MoS_2_ nanosheets with a large surface area were taken as the substrates, which can load abundant magnetic nanoparticles and peroxidase‐like nanoparticles (Au@Pt nanoflowers), providing magnetic separation capability and amplifying catalytic activity. Additionally, three metals (Pt, Au, and Ag) contained in these nanozymes further enhanced catalytic performance due to LSPR and the interaction of Pt/Au and Pt/Ag. Based on a magnetic separation and catalytic amplification system, 3D magnetic multi‐metallic nanozymes‐based LFIA can detect Flu A as low as 0.8 pg/mL within 26 min, 125 times lower than commercial colloidal gold‐based LFIA. Moreover, 20 clinical samples infected with Flu A were detected with an accuracy of 100%, and the sensitivity of inactive Flu A viruses was 140 copies/mL. This trifunctional LFIA integrates magnetic separation, colorimetric analysis, and catalytic amplification, exhibiting great potential in a pretreatment‐ and equipment‐free diagnostic method for rapid and accurate diagnosis of Flu A.

## Graphical abstract

The application of nanozymes in LFIA greatly improved the situation of insufficient colorimetric signals by catalytic reaction. 3D magnetic multi‐metallic nanozymes integrated the characteristics of great magnetism, enhanced colorimetric signal, and outstanding POD‐like activity, and were applied to LFIA for rapid and accurate detection of Influenza A viruses in throat swab samples.

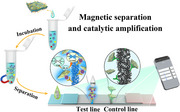

## 1 Introduction

Influenza (Flu) viruses usually cause upper respiratory tract infection in humans and are transmitted by direct contact or droplets through the respiratory route, including coughing, sneezing, talking, and so on. The typical symptoms of infected individuals were high fever, cough, headache, and other respiratory symptoms. According to the statistics, up to 650,000 people die annually because of the seasonal Flu. Even more, Flu is a common and highly pathogenic viral respiratory infection among children of all ages [1]. Moreover, Flu viruses can be divided into four types (A, B, C, and D) based on the antigenicity of nucleoprotein and matrix protein, but only Flu A can cause global pandemics as far as we know. Notably, the first influenza pandemic in the 21st century resulted from Flu A/H1N1 (2009), which spread to 214 countries and caused more than 18,000 deaths worldwide [2]. Early and accurate diagnosis of Flu A viruses is necessary for timely treatment of patients and effective control of the epidemic.

Recently, the lateral flow immunochromatographic assay (LFIA) has raised researchers’ increasing attention in the field of point‐of‐care testing (POCT). Due to the advantages of speed, low cost, simple operation, and limited sample treatment, LFIA was recognized as the optimum detecting method, especially in the resource‐limited field or developing countries [3]. However, qualitative or semi‐quantitative detection and limited sensitivity greatly hindered the extensive application of LFIA. Several technologies were introduced to LFIA to settle these shortcomings, such as fluorescence [4], surface‐enhanced Raman scattering [5], and electrochemistry [6]. However, the demand for detecting equipment and professional technicians still limits the application of LFIA, though the sensitivity has improved a lot. Moreover, the novel or improved nanomaterials, such as nanozymes, were prepared to improve the sensitivity of LFIA by enhancing the adsorbent capacity and signal of strips. Nanozymes, an artificial enzyme based on nanomaterials, are considered the major signal amplification method that utilizes enzyme‐catalytic reaction on the LFIA strips. The application of nanozymes greatly improved the situation of insufficient colorimetric signal of LFIA based on plasma nanomaterials (e.g., gold nanoparticles), which depended on their inherent optical properties [7]. Compared with the natural enzymes, nanozymes possessed similar catalytic performances, but more stable chemical properties, better biocompatibility, and lower cost. Notably, peroxidase (POD)‐like nanozymes can generate the hydroxyl radicals (•OH) and catalyze the conversion of chromogenic substrates into oxidative colored products, which were successfully applied for colorimetric and equipment‐free detection of bacteria [8, 9], viruses [10, 11], toxins [12, 13], proteins [14], and so on.

The catalytic performances of the nanozyme directly determined the sensitivity and accuracy of nanozyme‐based LFIA. To achieve highly sensitive detection of low levels of targets, researchers have dedicated themselves to seek nanozymes with excellent enzyme‐like activity. Nanomaterials used for synthesizing nanozymes mainly included metals (e.g., platinum, palladium) [15, 16], transition metal oxides (e.g., Fe_3_O_4_) [17, 18], covalent organic frameworks [19], metal organic frameworks [20, 21], and so on. Generally, nanozymes with a smaller size have higher catalytic activity due to the large specific surface area [22]. Nevertheless, nanoparticles with a smaller size and higher surface energy were unstable and tended to aggregate in the clinical complex samples, perhaps interfered by pH and salt ions in the samples [23]. Notably, studies have shown that the bi‐ or multi‐metallic nanozymes achieved higher synergistic catalytic performances than single‐metal nanozymes [24, 25]. The main mechanism is due to the addition of metals modulated the electronic structure of nanozymes, further enhancing the localized surface plasmon resonance (LSPR) effect, which can strengthen the catalytic efficiency [26]. Furthermore, the morphology of nanozymes significantly influences the catalytic efficiency. Two‐dimensional (2D) nanosheets and three‐dimensional (3D) frameworks exhibit great catalytic activity as they have more exposed surfaces and sites than common nanoparticles [27]. Some studies reported loading Fe_3_O_4_ nanoparticles on two‐dimensional MoS_2_ nanosheets by an in situ growth method to synthesize three‐dimensional multilayer structures, but it greatly relied on the adsorption capacity of MoS_2_ itself on the precursor, leading to low loading and uneven distribution of nanoparticles [28]. And some nanozymes requiring post‐synthesis annealing may generate agglomeration and size enlargement of nanoparticles due to the Ostwald ripening phenomenon, consequently reducing the specific surface area and compromising catalytic performance [29, 30]. Additionally, sample pretreatment is necessary for accurate diagnosis as the catalytic activity of nanozymes is easily influenced by the matrix effects in complex samples, which lead to false positive/negative results, such as serum, urine, and oropharyngeal/nasopharyngeal swabs [31]. Other causes for low sensitivity of LFIA were mainly owing to the limited target antigen in the sample and the limited immune‐labels captured by the test line of the LFIA strip [32]. Thus, further exploring strategies to synthesize high‐performance nanozymes is vital for enhancing the detection performance of LFIA.

In this study, we developed a novel 3D magnetic multi‐metallic nanozyme based on molybdenum disulfide (MoS_2_) nanosheets as immune‐labels to amplify the colorimetric signal and improve the detection sensitivity of LFIA in POCT. The 3D magnetic multi‐metallic nanozymes use 2D MoS_2_ nanosheets as the substrate, with satellite‐like Fe_3_O_4_/Ag nanoparticles and Au@Pt nanoflowers (AP NFs) as loaders. Fe_3_O_4_/Ag nanoparticles and AP NFs were sequentially and precisely assembled on 2D MoS_2_ nanosheets mediated by PEI, with a simple, fast, and economical process. Based on the large surface area of the 3D multilayer structure, the loading number of functional nanoparticles and specific capture antibodies was greatly improved, further enhancing the catalytic activity of nanozymes. Specifically, Fe_3_O_4_/Ag nanoparticles not only provided magnetism for the 3D magnetic nanozyme, but Ag could further enhance the catalytic performance of 3D magnetic nanozyme due to the LSPR between metals [33]. Furthermore, the 3D magnetic multi‐metallic nanozyme integrated the characteristics of strong magnetism, enhanced colorimetric signal, and outstanding POD‐like activity, avoiding the impurities in the complex samples from interfering with the catalytic performance of nanozymes.

To demonstrate the ultrasensitive and excellent catalytic performance of the 3D magnetic multi‐metallic nanozyme, MoS_2_@Fe_3_O_4_/Ag@AP was used to synthesize the immune labels to specifically recognize and capture Flu A in clinical complex samples. Under the superior magnetic and catalytic performances of MoS_2_@Fe_3_O_4_/Ag@AP, we present trifunctional LFIA strips based on 3D magnetic multi‐metallic nanozyme for Flu A detection in clinical complex samples. Additionally, we choose non‐toxic TMB as the chromogenic substrate to amplify the colorimetric signals of LFIA strips, rather than other carcinogenic chromogenic substrates such as DAB. With the specific conjugation of antigen‐antibody and magnetic separation, the proposed trifunctional LFIA strips can detect Flu A with a sensitivity of 0.8 pg/mL, which is 125 times lower than that of commercial colloidal gold‐based LFIA strips. Moreover, the throat swab samples from patients infected with Flu A and inactive Flu A viruses were also used to testify the accuracy and validity of the as‐prepared trifunctional LFIA strips. Above all, the proposed 3D magnetic multi‐metallic nanozyme‐based LFIA can serve as a novel and efficient signal amplification strategy in POCT, and this work expands new directions for thinking about the design of metal nanozymes for POCT applications.

## 2 Experimental Section

### 2.1 Materials and Instruments

Materials and instruments used in this study are described in Supporting Information S1.

### 2.2 Synthesis of the 3D Magnetic Multi‐Metallic Nanozymes

The 3D magnetic multi‐metallic nanozymes, namely MoS_2_@Fe_3_O_4_/Ag@AP, were synthesized by the PEI‐mediated layer‐by‐layer assembly method. First, Fe_3_O_4_/Ag nanoparticles were synthesized by a one‐pot reaction [34]. Au@Pt nanoflowers (AP NFs) were prepared by a previously reported AA‐mediated seed growth method with some modifications [35]. The detailed preparation steps of Fe_3_O_4_/Ag nanoparticles and AP NFs are illustrated in Supporting Information S2. Briefly, 5 mg of MoS_2_ nanosheets was dispersed in 10 mL of distilled water under sonication for 10 min. The smaller MoS_2_ nanosheets were discarded by centrifugation at 7000 rcf for 8 min. The sediment was resuspended by 10 mL of PEI (1 mg/mL) aqueous solution and sonicated for 40 min to prepare the first layer of positively charged PEI on the surface of MoS_2_ (MoS_2_/PEI). The synthesized MoS_2_/PEI was washed with deionized water twice by centrifugation (7000 rcf for 8 min) and resuspended by 10 mL of deionized water. Then, 0.5 mL of the as‐prepared Fe_3_O_4_/Ag (5 mg/mL) was added to the above MoS_2_/PEI solution and continued to ultrasonic for 30 min. The negatively charged Fe_3_O_4_/Ag could adsorb on the surface of MoS_2_/PEI by electrostatic forces to form MoS_2_@Fe_3_O_4_/Ag. The prepared MoS_2_@Fe_3_O_4_/Ag was washed with deionized water twice by magnetic enrichment. Next, the second layer of PEI was adsorbed on the MoS_2_@Fe_3_O_4_/Ag by the same operation as the first layer. After washing with deionized water, the synthesized MoS_2_@Fe_3_O_4_/Ag/PEI was resuspended in 10 mL of water. 4 mL of AP NFs was then added to them and sonicated for another 30 min. Finally, the prepared MoS_2_@Fe_3_O_4_/Ag@AP was washed twice with deionized water and stored in 5 mL of ethanol.

### 2.3 Preparation of 3D Magnetic MoS_2_@Fe_3_O_4_/Ag@AP Immune Labels

MUA was introduced to the 3D magnetic MoS_2_@Fe_3_O_4_/Ag@AP to provide the carboxyl sites that can bind to antibodies. Briefly, 80 µL of MUA ethanol solution (10 mM) was added to 1 mL of MoS_2_@Fe_3_O_4_/Ag@AP and sonicated for 1 h. After magnetic enrichment, the sediments were resuspended in 500 µL of MES buffer (100 mM, pH 5.5) and sonicated for several seconds to make the dispersion uniform. Then, the mixture of EDC and NHS (1 mM/2 mM) was added to the above solution to activate MoS_2_@Fe_3_O_4_/Ag@AP under sonication for 15 min. The activated MoS_2_@Fe_3_O_4_/Ag@AP was then separated by the external magnet and resuspended in 200 µL of PBST (10 mM, 0.05 wt% Tween‐20). Next, 10 µg of anti‐Flu A antibodies was added and incubated for 2.5 h by vigorously vibrating at room temperature. The mixture was injected with 100 µL of 10 wt% BSA to block the uncombined sites and incubated for another 1 h. After washing with 1 mL of PBST, the 3D magnetic immune labels were finally stored in 200 µL of preservation solution (10 mM PBS containing 1 wt% BSA, 0.5 wt% sucrose, and 0.02 wt% NaN_3_). The assembly of the LFIA strip was described in detail in the Supporting Information S4.

### 2.4 Detection of Flu A via the 3D Magnetic Multi‐Metallic Nanozymes‐Based LFIA

To analyze the sensitivity of this method, a series of different concentrations (0.001–50 ng/mL) of Flu A nucleoprotein was prepared as a standard sample. First, 1 mL of Flu A nucleoprotein dilution was added to the tube, followed by 1.2 µL of 3D magnetic immune labels. After 10 min of incubation, the complex of Flu A nucleoprotein‐labels was magnetically enriched and resuspended with 70 µL of loading buffer (10 mM PBS containing 1 wt% Tween‐20 and 1 wt% BSA). Next, the mixture was all dropped onto the sample pad of the as‐prepared LFIA strip. After 15 min of chromatographic reaction, 1 µL of chromogenic solution (10 µL of 50 mM HAc, 1 µL of 4 mM H_2_O_2_, and 1 µL of 2 mM TMB) was dripped on the test line. And the colorimetric signals of the test line were greatly enhanced after 30 s. Notably, the colorimetric signals can be both qualified by the naked eye and quantified with the help of a smartphone. In short, the photographs of strips were captured by the smartphone, and the gray intensity of strips was then analyzed using ImageJ software. All photographs of strips were acquired under fixed ambient lighting and constant camera‐to‐strip distance to ensure measurement consistency. The average value of gray intensity within the test line was regarded as the optical intensity of this strip. Each experiment was performed three times.

### 2.5 Detection of Inactivated Flu A Virus and Clinical Throat Swab Samples

To prove the clinical applicability of this method, inactivated viruses and 20 clinical samples were tested by the 3D magnetic multi‐metallic nanozymes‐based LFIA strips. Notably, Flu A viruses (H1N1 2009/A) were cultured in chick embryos and quantified by PCR in our laboratory, and the clinical throat swab samples infected with Flu A were collected from Capital Institute of Pediatrics with the approval of the Ethics Committee of Capital Institute of Pediatrics (registration number: SHERLL‐2024‐033). Written informed consent was obtained from all patients.

Firstly, the inactivated Flu A viruses were added to 1 mL of lysis solution and gradient diluted with the lysis solution. Then, 1.2 µL of immune labels was added to capture the virus nucleoprotein and form the complex of nucleoprotein‐labels. Similarly, the inactivated virus solution and immune labels were incubated for 15 min, enriched, and resuspended in the loading buffer. The chromatographic and chromogenic processes on the strips were the same as those described above. The clinical samples were first diluted 10 times with lysis solution and incubated with 1.2 µL of the immune labels for 15 min. Other detecting steps were as described above. Photographs of the tested strips were recorded by the smartphone and analyzed by the ImageJ software.

## 3 Results and Discussions

### 3.1 Detection Principle of 3D Magnetic Multi‐Metallic Nanozymes‐Based LFIA

**FIGURE 1:**
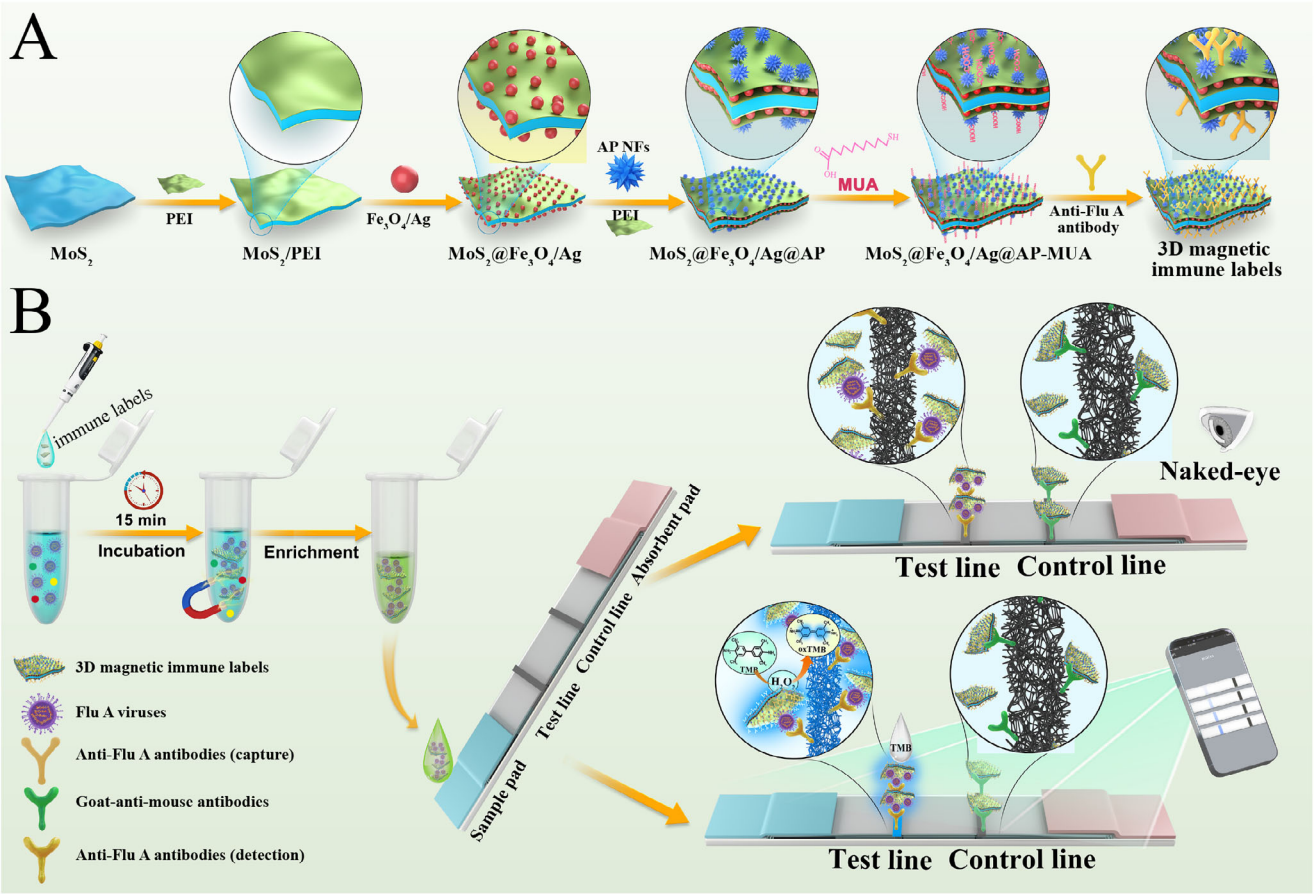
Schematic of 3D magnetic multi‐metallic nanozymes‐based LFIA for Flu A detection. (A) Preparation of MoS_2_@Fe_3_O_4_/Ag@AP‐based immune‐labels. (B) Magnetic separation and catalytic amplification system via 3D magnetic multi‐metallic nanozymes‐based LFIA.

The synthesis of 3D magnetic MoS_2_@Fe_3_O_4_/Ag@AP is illustrated in Figure 1. In brief, MoS_2_ nanosheets were used as the two‐dimensional substrate for layer‐by‐layer assembly with the help of PEI, as shown in Figure 1A. Fe_3_O_4_/Ag nanoparticles and AP NFs were firstly prepared by redox and AA‐mediated reduction methods, respectively. Fe_3_O_4_/Ag nanoparticles and AP NFs were successively adsorbed on the two‐dimensional MoS_2_ nanosheets under sonication. Notably, AgNO_3_ was not only used as the oxidant during the process of preparing Fe_3_O_4_/Ag nanoparticles, but its oxidation product (Ag) concomitantly strengthened the catalytic activity of AP NFs in MoS_2_@Fe_3_O_4_/Ag@AP due to LSPR [36]. Similarly, the core of AP NFs, Au, can both provide growth sites for the Pt shell and further improve the POD‐like activities of Pt. Then, anti‐Flu A antibodies were modified on the 3D magnetic MoS_2_@Fe_3_O_4_/Ag@AP to prepare functional immune labels. The testing process of magnetic separation and catalytic amplification system via 3D magnetic multi‐metallic nanozymes‐based LFIA is shown in Figure 1B. In detail, the as‐prepared immune‐labels were injected into the collected throat swab sample to specifically recognize and capture Flu A in the sample, obtaining the complexes of immune labels‐Flu A. After 10 min of incubation, the formed complexes were magnetically enriched and then added to the trifunctional LFIA strips for Flu A detection. The antibodies precoated on the test line can specifically capture the immune labels‐virus complex and form a labels‐virus‐antibodies sandwich structure. After the chromatographic reaction, the chromogenic solution was dropped on the test lines of LFIA strips to amplify the colorimetric signal due to the catalytic performances of MoS_2_@Fe_3_O_4_/Ag@AP. The strengthened visual signals were captured by the smartphone and analyzed by the ImageJ software for quantitative detection of Flu A.

### 3.2 Characterization of 3D Magnetic MoS_2_@Fe_3_O_4_/Ag@AP

**FIGURE 2:**
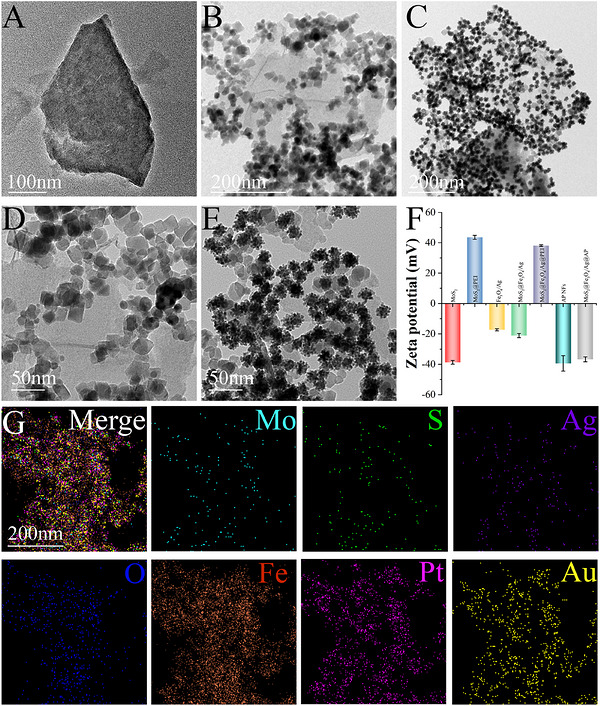
HR‐TEM images of MoS_2_ nanosheets (A), MoS_2_@Fe_3_O_4_/Ag (B), and (C) MoS_2_@Fe_3_O_4_/Ag@AP. The magnified HR‐TEM images of MoS_2_@Fe_3_O_4_/Ag (D) and MoS_2_@Fe_3_O_4_/Ag@AP (E). (F) Zeta potentials of the MoS_2_@Fe_3_O_4_/Ag@AP at different stages. (G) EDS mapping images of MoS_2_@Fe_3_O_4_/Ag@AP.

The high resolution‐transmission electron microscopy (HR‐TEM) image of MoS_2_ nanosheets is shown in Figure 2A. First, the thin MoS_2_ nanosheets (400–800 nm) were selected by centrifugation and coated with the first layer of PEI to attach Fe_3_O_4_/Ag magnetic nanoparticles. The negatively charged MoS_2_ nanosheets were transformed into positively charged ones due to the existence of PEI with a positive charge. Thus, positively charged MoS_2_/PEI can adsorb Fe_3_O_4_/Ag magnetic nanoparticles (30 nm) with a negative charge. Figure 2B,D indicates that numerous small Fe_3_O_4_/Ag magnetic nanoparticles were attached to the substrate of MoS_2_ nanosheets. Next, the second layer of PEI was coated on the surface of MoS_2_@Fe_3_O_4_/Ag to adsorb AP NFs with the properties of nanozymes. As shown in Figure 2C, the surface of MoS_2_@Fe_3_O_4_/Ag was full of negatively charged AP NFs due to the electrostatic adsorption. The magnified HR‐TEM image of MoS_2_@Fe_3_O_4_/Ag@AP indicated that AP NFs were not only attached direct on the surface of MoS_2_ but also to the Fe_3_O_4_/Ag nanoparticles (Figure 2E). In addition, the zeta potentials of the products during different stages were recorded as shown in Figure 2F. It can be seen that the zeta potential of MoS_2_, Fe_3_O_4_/Ag, and AP NFs were all negative, namely −38.7, −17.3, and −39.4 mV. Notably, the zeta potential of MoS_2_/PEI rocketed from −38.7 to 43.6 mV while the first layer of PEI was coated on MoS_2_, but decreased to −21.1 mV after the affiliation of Fe_3_O_4_/Ag. Furthermore, the zeta potential of MoS_2_@Fe_3_O_4_/Ag/PEI was 38.1 mV after the cover of the second layer of PEI and reduced to −36.7 mV as MoS_2_@Fe_3_O_4_/Ag@AP was formed. This regular change in zeta potential exactly confirmed the successful synthesis of MoS_2_@Fe_3_O_4_/Ag@AP. Additionally, the energy dispersive spectroscopy (EDS) mapping images and the element distribution data of MoS_2_@Fe_3_O_4_/Ag@AP demonstrated the distribution and contents of Mo, S, Fe, O, Ag, Au, and Pt atoms, suggesting that Fe_3_O_4_/Ag and AP NFs were successfully assembled on MoS_2_ (Figure 2G and Figure S1).

**FIGURE 3:**
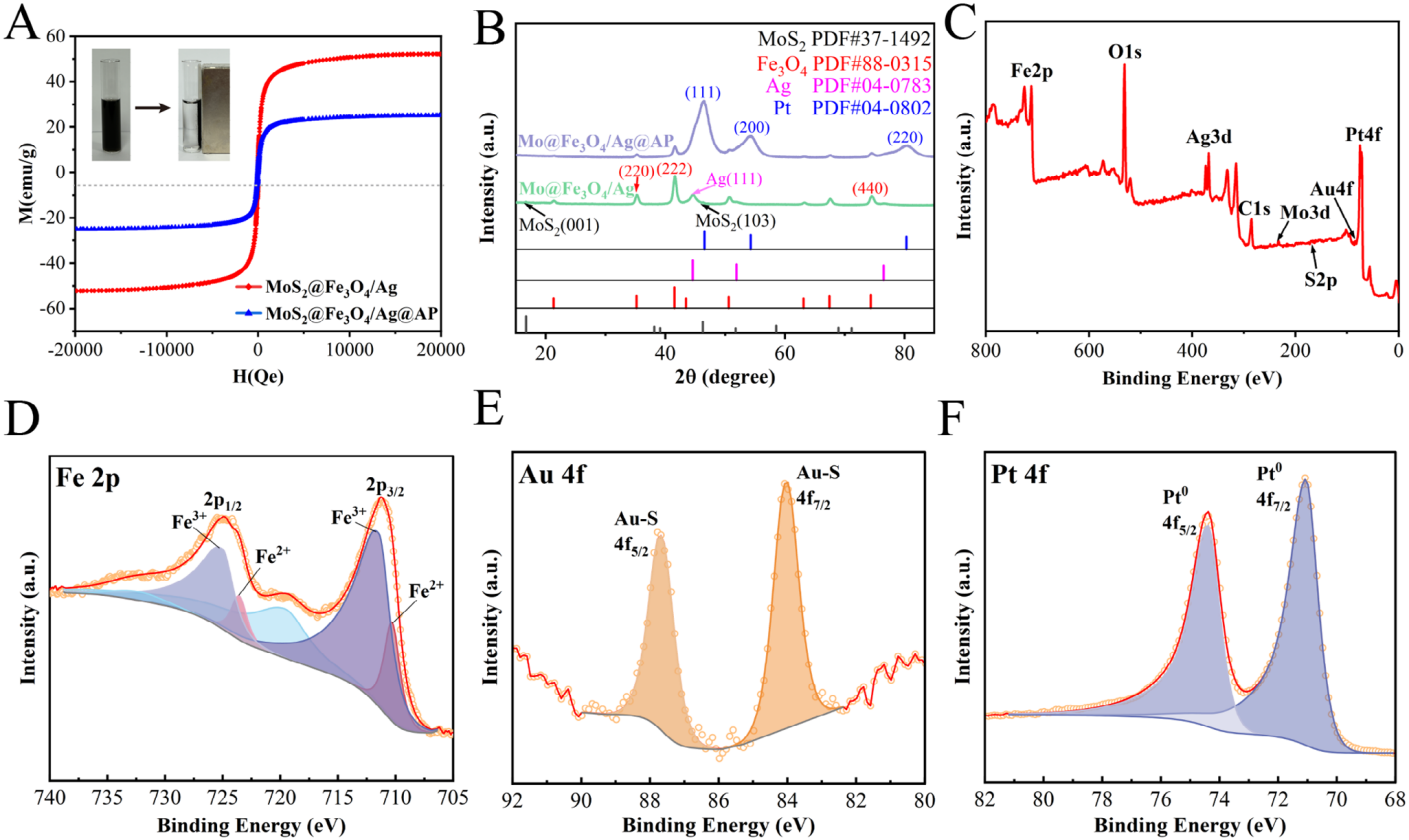
(A) Magnetic hysteresis curves of MoS_2_@Fe_3_O_4_/Ag and MoS_2_@Fe_3_O_4_/Ag@AP. (B) XRD patterns of MoS_2_@Fe_3_O_4_/Ag and MoS_2_@Fe_3_O_4_/Ag@AP. (C) Wide‐scan XPS patterns of MoS_2_@Fe_3_O_4_/Ag@AP. Corresponding high‐resolution (D) Fe 2p, (E) Au 4f and (F) Pt 4f patterns of MoS_2_@Fe_3_O_4_/Ag@AP.

The magnetic properties of the as‐prepared composites were also measured. As revealed in Figure 3A, the magnetization saturation (*M*_S_) value of MoS_2_@Fe_3_O_4_/Ag was 52.15 emu g^−1^. But the *M*_S_ value of MoS_2_@Fe_3_O_4_/Ag @AP was greatly decreased to 25.05 emu g^−1^ due to the dense adsorption of AP NFs with non‐magnetic elements. The photograph inset in Figure 3A shows that the synthesized MoS_2_@Fe_3_O_4_/Ag@AP can be separated from the solution by the external magnet within 40 s. Moreover, the X‐ray diffraction (XRD) patterns of MoS_2_@Fe_3_O_4_/Ag and MoS_2_@Fe_3_O_4_/Ag@AP were measured to analyze the structure and crystalline phase, as shown in Figure 3B. The diffraction peaks of MoS_2_@Fe_3_O_4_/Ag and MoS_2_@Fe_3_O_4_/Ag@AP can be indexed to the corresponding Powder Diffraction File (PDF) database. Specifically, the main peaks of the green curve (MoS_2_@Fe_3_O_4_/Ag) shown in Figure 3B at 16.71° and 46.25° were indexed to the corresponding reflections of the (001) and (103) crystalline planes of MoS_2_ (PDF#37‐1492) [37]. The main peaks at 35.17°, 41.49°, and 74.34° correspond to the reflections of the (220), (222), and (440) crystalline planes of Fe_3_O_4_ (PDF#88‐0315), respectively, while the main peak at 44.57° corresponds to the reflections of (111) crystalline plane of Ag (PDF#04‐0783) [34]. Furthermore, the main peaks of the purple curve (MoS_2_@Fe_3_O_4_/Ag@AP) at 46.52°, 54.26°, and 80.3° correspond to the reflections of the (111), (200), and (220) crystalline planes of Pt (PDF#04‐0802), respectively [38].

Additionally, the X‐ray photoelectron spectroscopy (XPS) patterns shown in Figure 3C illustrate that the elements of Mo, S, Fe, O, Ag, Au, and Pt coexisted in the MoS_2_@Fe_3_O_4_/Ag@AP composite. Particularly, the peaks of high‐resolution Fe pattern at 710.29 and 723.56 eV correspond to 2p_3/2_ and 2p_3/2_ of Fe^2+^ (17.93%), and the peaks at 711.44 and 725.02 eV corresponded to 2p_3/2_ and 2p_3/2_ of Fe^3+^ (82.07%), as shown in Figure 3D. In addition, the peaks at high‐resolution Au pattern at 84.02 and 87.65 eV were the features of 4f_7/2_ and 4f_5/2_ of Au (Figure 3E). Similarly, the peaks at 71.05 and 74.39 eV shown in Figure 3F correspond to 4f_7/2_ and 4f_5/2_ of Pt^0^ (100%), suggesting the catalytic property of MoS_2_@Fe_3_O_4_/Ag@AP was excellent since valence state of Pt in it was all 0. Above structural characterizations of the synthesized materials all confirmed the successful formation of MoS_2_@Fe_3_O_4_/Ag@AP.

### 3.3 Catalytic Properties of 3D Magnetic Multi‐Metallic Nanozymes

**FIGURE 4:**
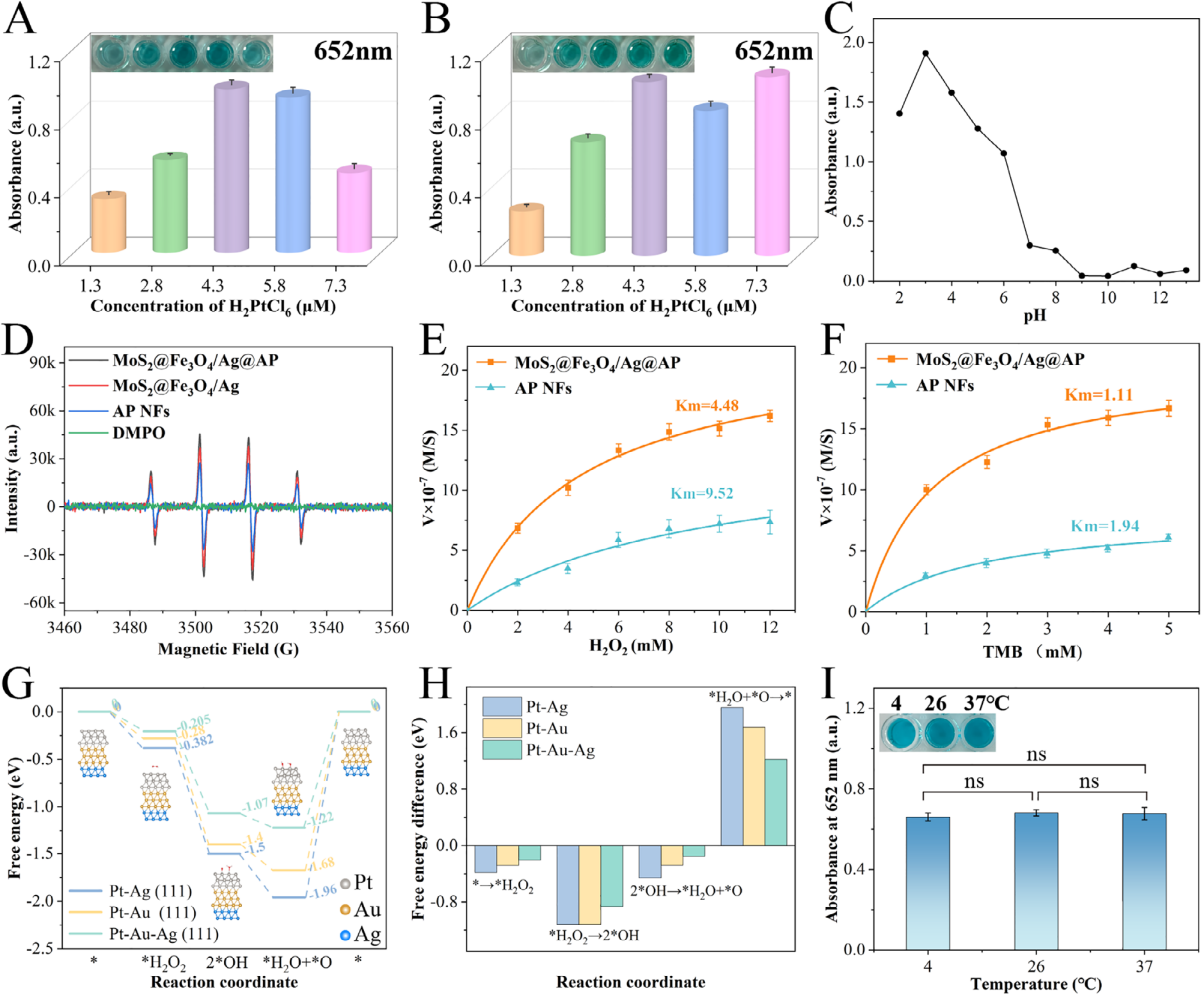
(A) Absorbance (652 nm) of AP NFs with different concentrations of H_2_PtCl_6_: 1.3, 2.8, 4.3, 5.8, and 7.3 µM. (B) Absorbance (652 nm) of MoS_2_@Fe_3_O_4_/Ag@AP prepared by AP NFs with corresponding concentrations of H_2_PtCl_6_ shown in (A). (C) Absorbance (652 nm) of MoS_2_@Fe_3_O_4_/Ag@AP measured in different pH values of chromogenic solution. (D) ESR spectra of AP NFs, MoS_2_@Fe_3_O_4_/Ag, and MoS_2_@Fe_3_O_4_/Ag@AP in the DMPO system. Steady‐state kinetic analysis of MoS_2_@Fe_3_O_4_/Ag@AP and AP NFs by different concentrations of H_2_O_2_ (E) and TMB (F). Error bars are calculated from three separate experiments. Free energy change profiles (G) and the corresponding free energy difference (H) of TMB oxidation reaction pathways on the surface of three catalytic models. (I) Catalytic properties of MoS_2_@Fe_3_O_4_/Ag@AP stored at different temperatures (4°C, 26°C, and 37°C). The inset shows the color of the corresponding solutions after the catalytic reaction.

For the excellent catalytic performance of the synthesized MoS_2_@Fe_3_O_4_/Ag@AP, different thicknesses of Pt shell coated on the Au NPs were firstly prepared. As shown in Figure 4A, the absorbance values at 652 nm increased with the increasing concentration of H_2_PtCl_6_, but decreased when the concentration of H_2_PtCl_6_ was higher than 4.3 µM. Similarly, the average diameter of AP NFs increased from 15.06 to 28.3 nm while the concentration of H_2_PtCl_6_ increased from 0 to 4.3 µM (Figure S2). However, the average diameter of AP NFs grew to 26.5 nm (5.8 µM) and 27.38 nm (7.3 µM). In addition, the UV–vis spectra of AP NFs with different thicknesses of Pt shell were investigated and nearly did not change while the addition concentration of H_2_PtCl_6_ was lower than 4.3 µM (Figure S3). Furthermore, these AP NFs with different thicknesses of Pt shell were used to synthesize MoS_2_@Fe_3_O_4_/Ag@AP. The UV–vis absorbance peak (652 nm) of MoS_2_@Fe_3_O_4_/Ag@AP in the H_2_O_2_/TMB system increased with the concentration of H_2_PtCl_6_, increasing from 1.3 to 4.3 µM, but the trend slowed down when the concentration of H_2_PtCl_6_ exceeded 4.3 µM (Figure 4B and Figure S4). Therefore, 4.3 µM of H_2_PtCl_6_ was selected to grow a Pt shell on the Au NPs for the following experiments. UV–vis spectra of MoS_2_@Fe_3_O_4_/Ag@AP at different stages were also evaluated (Figure S5). Obviously, the absorbance peak of MoS_2_ and MoS_2_@Fe_3_O_4_/Ag nearly did not change after the addition of PEI, but changed significantly after the absorption of Fe_3_O_4_/Ag and AP NFs on MoS_2_ nanosheets. The increasing absorbance of MoS_2_@Fe_3_O_4_/Ag@AP at different stages indicated their colorimetric ability gradually enhanced.

The pH of the chromogenic solution greatly influenced the detection sensitivity based on nanozymes. Figure 4C illustrates that the 3D magnetic MoS_2_@Fe_3_O_4_/Ag@AP exhibited perfect POD‐like property ranging from pH 2 to 6, especially highest at pH 3. Therefore, the pH of chromogenic solution (TMB/H_2_O_2_ detection system) was adjusted to 3 to keep the best POD‐like performance of the 3D magnetic MoS_2_@Fe_3_O_4_/Ag@AP. Moreover, the electron spin resonance (ESR) of AP NFs, MoS_2_@Fe_3_O_4_/Ag, and MoS_2_@Fe_3_O_4_/Ag@AP were also investigated to analyze the POD‐like performance. As shown in Figure 4D, MoS_2_@Fe_3_O_4_/Ag@AP displayed the highest reactive oxygen species (ROS) (·OH) in the 5, 5‐dimethyl‐1‐pyrroline N‐oxide (DMPO) system, suggesting the distinguished POD‐like properties of MoS_2_@Fe_3_O_4_/Ag@AP. The steady‐state kinetic analysis for POD‐like property of MoS_2_@Fe_3_O_4_/Ag@AP and AP NFs was also measured with TMB and H_2_O_2_ detection system, respectively, using the Michaelis–Menten model (Figure 4E,F). Specifically, the typical steady‐state kinetic curves were plotted by designed concentration of the substrates (H_2_O_2_ and TMB) and corresponding absorbance at 652 nm over time. By calculation, the Michaelis‐Menten constant (*K*_m_) values of MoS_2_@Fe_3_O_4_/Ag@AP were 4.48 and 1.11 mM for H_2_O_2_ and TMB, respectively, suggesting the higher affinity of MoS_2_@Fe_3_O_4_/Ag@AP for TMB. AP NFs exhibited substantially elevated *K*_m_ values (9.52 mM for H_2_O_2_ and 1.94 mM for TMB), both higher than those of MoS_2_@Fe_3_O_4_/Ag@AP, suggesting their inferior substrate affinity and catalytic efficiency. Additionally, the corresponding maximum reaction velocities (*V*_max_) were 2.25 and 2.04 × 10^−6^ M/S for H_2_O_2_ and TMB, respectively, indicating a higher catalytic rate. The values of *K*_m_ and *V*_max_ for MoS_2_@Fe_3_O_4_/Ag@AP, HRP, and other related POD‐like nanozymes were listed in Table S1. By comparison, MoS_2_@Fe_3_O_4_/Ag@AP showed greater catalytic properties than HRP, and most reported POD‐like nanozymes.

In addition, density functional theory (DFT) calculations were performed by following four steps to verify the mechanism of enhanced peroxidase‐like activity of the 3D magnetic nanozymes (Figure 4G). As the MoS_2_@Fe_3_O_4_ substrate was the same in different nanozymes, Pt‐Ag, Pt‐Au, and Pt‐Au‐Ag were used for DFT calculation on the H_2_O_2_ decomposition to illustrate the effect of NPs on the catalytic efficiency of peroxidase catalysis of 3D nanozymes. As shown in Figure 4G, the rate‐determining step for these three catalytic models was the fourth step (*H_2_O+*O→*), the easiest step in the catalytic circle of the 3D magnetic nanozymes. As the rate‐determining step was ascending, the smaller the energy difference, the easier it was to generate. Specifically, the energy barrier of Pt‐Au‐Ag (111) was highest (1.96 eV) while that of Pt‐Ag (111) was lowest (1.22 eV), revealing that the generation of ·OH was less hindered in Pt‐Au‐Ag than in Pt‐Ag and Pt‐Au. The free energy difference in each TMB oxidation reaction step further confirmed that the structural performance of the Pt‐Au‐Ag model was optimal, revealing that the catalytic performance of the MoS_2_@Fe_3_O_4_/Ag@AP was excellent (Figure 4H). Additionally, we compared the POD‐like property of the 3D magnetic nanozyme without Au and Ag by the TMB/H_2_O_2_ system, and the results revealed that MoS_2_@Fe_3_O_4_/Ag@AP was the best (Figure S6). Moreover, we evaluated the stability of MoS_2_@Fe_3_O_4_/Ag@AP under various conditions as follows. The POD‐like activity of MoS_2_@Fe_3_O_4_/Ag@AP was highly reproducible with the relative standard deviation (RSD) value of 2.7% by evaluating four batches of MoS_2_@Fe_3_O_4_/Ag@AP for TMB (Figure S7). Furthermore, the MoS_2_@Fe_3_O_4_/Ag@AP maintained great and stable catalytic activity stored at different temperatures and after storage for 60 days (Figure 4I and Figure S7). As the salt in the samples can compromise nanozyme performance, the salt stability of the synthesized MoS_2_@Fe_3_O_4_/Ag@AP was evaluated by monitoring the UV–vis in NaCl solutions (0–1 M), exhibiting excellent dispersibility and physical stability (Figure S8).

### 3.4 3D Magnetic Multi‐Metallic Nanozymes‐Based LFIA for Ultrasensitive Detection of Flu A

MoS_2_@Fe_3_O_4_/Ag@AP was confirmed to be excellent enrichment and catalytic performances through the above results. Therefore, 3D magnetic MoS_2_@Fe_3_O_4_/Ag@AP was used to prepare immune labels to capture and enrich Flu A viruses from clinical throat swab samples without pretreatment. First, successful modifications of MUA and antibodies were confirmed with zeta potential and Fourier‐transform spectroscopy (FTIR). As shown in Figure S9A, the zeta potential was gradually decreased with the addition of MUA and antibodies. Infrared spectra of the MoS_2_@Fe_3_O_4_/Ag@AP and corresponding immune labels were measured by Fourier‐transform spectroscopy (FTIR). As shown in Figure S9, the FTIR spectra of antibody and immune labels both have characteristic absorption peaks at 1641 and 1530 cm^−1^, which correspond to the protein amide bands I and II, respectively [39], revealing the successful conjugation of antibodies. The immune labels can specifically recognize Flu A viruses and are conjugated to form a label‐virus complex. And the formed complex can be separated from the impurities in the samples by magnetic enrichment. By use of the POD‐like property of MoS_2_@Fe_3_O_4_/Ag@AP, the chromogenic solution (TMB/H_2_O_2_ system) turned white to blue on the test line of strips since TMB was oxidized to oxTMB in the presence of H_2_O_2_. Therefore, the light visual color or colorless on the test line can be greatly enhanced with the chromogenic solution, especially for lower concentrations of viruses in the sample.

For the ultrasensitive detection of Flu A via 3D magnetic MoS_2_@Fe_3_O_4_/Ag@AP‐based LFIA, several key parameters during the detection steps were optimized. First, chromogenic solutions based on TMB substrate from different companies were tested to choose the optimal chromogenic solution. As shown in Figure S10, compared with other chromogenic solutions purchased from ZOMANBIO, Sigma, and Biopanda, the TMB/H_2_O_2_ system made by ourselves displayed a more homogeneous test line. Therefore, we took the TMB/H_2_O_2_ system made by ourselves as a signal amplification solution. The components and corresponding content of the loading buffer were first optimized to avoid nonspecific adsorption and improve the detection sensitivity. By observing Figure S11, 1 wt% PBST containing 1 wt% BSA was the optimal loading buffer with the highest signal‐to‐noise ratio. Additionally, the concentration of antibodies on the test line was studied, and the ideal concentration was 1 mg/mL (Figure S12). Similarly, 1.2 µL of immune labels was enough to capture viruses from the sample shown in Figure S13.

**FIGURE 5:**
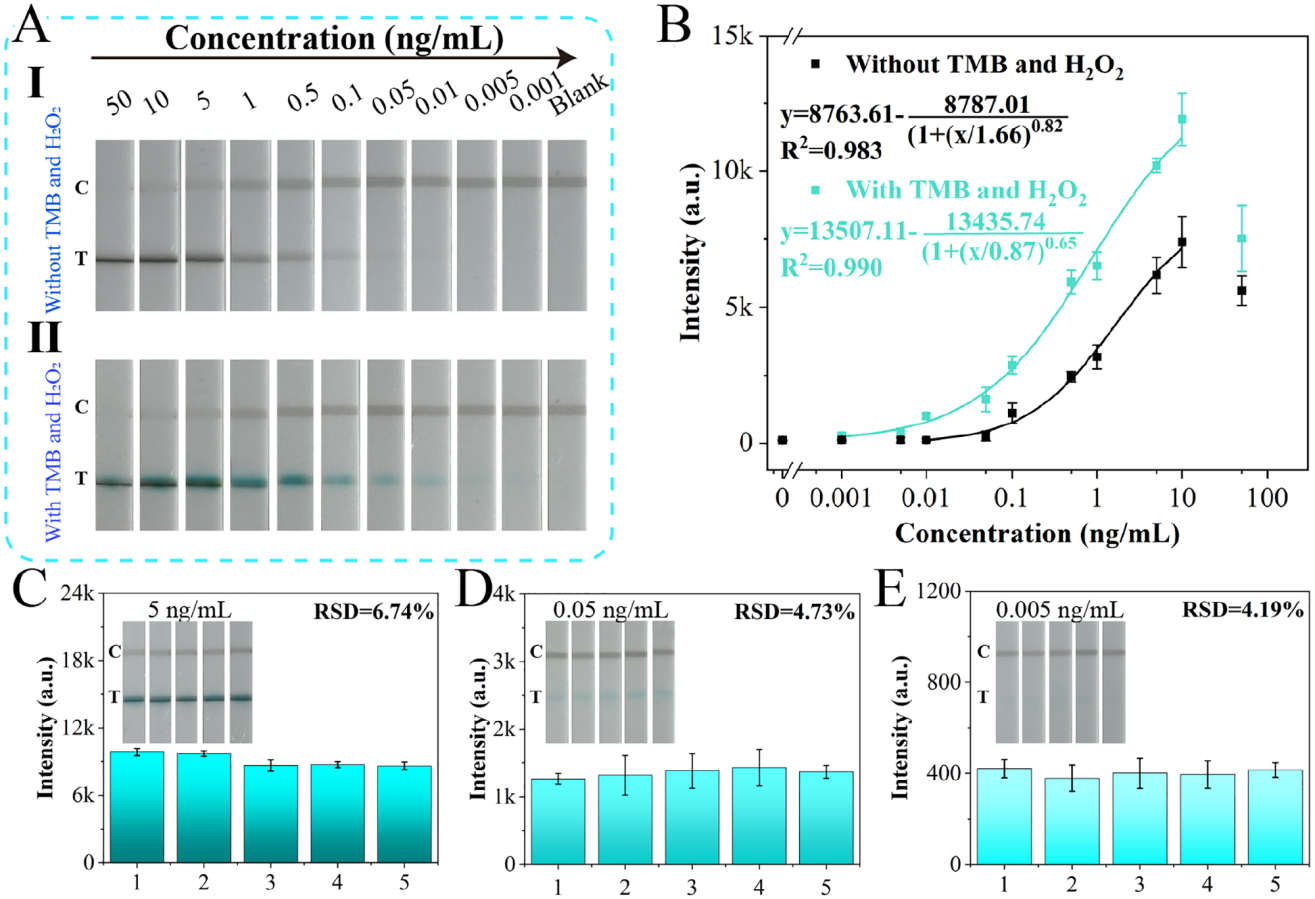
(A) Photographs of 3D magnetic MoS_2_@Fe_3_O_4_/Ag@AP‐based trifunctional LFIA strips for different gradient concentrations of Flu A nucleoprotein before (I) and after (II) catalytic reaction. (B) Calibration curves for Flu A nucleoprotein according to the detection results in (A). Photographs (inset) and corresponding gray values on test lines of five repeated tests for Flu A with concentrations of 5 (C), 0.05 (D), and 0.005 (E) ng/mL via the MoS_2_@Fe_3_O_4_/Ag@AP‐based LFIA strips. Error bars are calculated from three separate experiments.

Under the optimal conditions, we established a trifunctional LFIA based on 3D magnetic multi‐metallic nanozymes for ultrasensitive detection of Flu A, integrating the characteristics of magnetism, colorimetric analysis, and catalytic amplification. A series of different concentrations of Flu A nucleoprotein was prepared and used to test the sensitivity and quantitative property of this trifunctional LFIA strip. As shown in Figure 5A, the visible test line by naked eyes before and after the drop of chromogenic solution (TMB/H_2_O_2_ system) was 0.05 ng/mL and 5 pg/mL, respectively, improving 10 times. Compared with others, the test line corresponding to 50 ng/mL was relatively thin since the immune labels were mostly gathered at the bottom. Furthermore, the original and strengthened signals of the test line by catalysis were both analyzed by the ImageJ software. The gray values of the test line and corresponding concentration (0.001–50 ng/mL) of standard antigen (Flu A nucleoprotein) were used to plot the calibration curves for Flu A (Figure 5B). The limit of detection (LOD) for Flu A nucleoprotein before and after catalytic reaction via the trifunctional LFIA strips was calculated to be 0.01 and 0.8 pg/mL, respectively (defined as y_blank _+ 3 × SD_blank_, y_blank_ is the mean intensity of the blank and SD_blank_ is the standard deviation of the blank). In addition, the performances of AP NFs, MoS_2_@AP, and MoS_2_@Fe_3_O_4_/Ag@AP‐based trifunctional LFIA strips were evaluated as shown in Figure S14. As shown in Figure S14B, the color of test lines corresponding to the low concentration of Flu A nucleoprotein using MoS_2_@Fe_3_O_4_/Ag@AP‐based immune labels was deeper than that using MoS_2_@AP and AP NFs‐based immune labels. According to the gray values of the corresponding test lines (Figure S14C), the sensitivity of MoS_2_@Fe_3_O_4_/Ag@AP‐based LFIA strips was 0.8 pg/mL, 10 and 100 times lower than that of MoS_2_@AP‐based LFIA (10 pg/mL) and AP‐based LFIA (100 pg/mL). Furthermore, commercial colloidal gold‐based LFIA strips from three different companies (Biodragon, Easy Quarter, and Runbo Fude) were used to detect the same concentration of Flu A nucleoprotein (Figure S15). The photographs indicated that the visual sensitivities of these three commercial strips were both 100 pg/mL, 125 times higher than our proposed trifunctional LFIA strips. All the above results suggested the high sensitivity and quantitative performances of the 3D magnetic multi‐metallic nanozymes‐based LFIA for the detection of Flu A.

Moreover, the stability of the proposed 3D magnetic multi‐metallic nanozymes‐based LFIA strips was verified as follows. Figure 5C–E reveals that the gray values on the test lines were similar in each group. The RSD values were calculated to be 6.74%, 4.73%, and 4.19% for 5, 0.05, and 0.005 ng/mL of Flu A nucleoprotein, respectively, suggesting the high reproducibility of this method. Additionally, 0.01 and 2 ng/mL of Flu A nucleoprotein were used to evaluate the detection performance of MoS_2_@Fe_3_O_4_/Ag@AP‐based LFIA strips stored at different temperatures. As shown in Figure S17, the gray values of the strips before and after catalytic reaction were analyzed by ImageJ software, and the results showed that the intensities of the strips under different temperatures were consistent, indicating the superior stability of MoS_2_@Fe_3_O_4_/Ag@AP‐based LFIA strips. We also detected different concentrations of Flu A nucleoprotein via three batches of LFIA strips (Figure S18). Based on the calibration curves for Flu A nucleoprotein shown in Figure S18(III), the LODs for Flu A nucleoprotein before and after catalytic reaction were calculated and shown in Table S2. The RSD values of the sensitivity of these three batches of LFIA strips were 9.09% and 14.78% for before and after the catalytic reaction, respectively, revealing the excellent reproducibility of the trifunctional LFIA based on 3D magnetic multi‐metallic nanozymes.

### 3.5 Application in Clinical Throat Swab Samples

**FIGURE 6:**
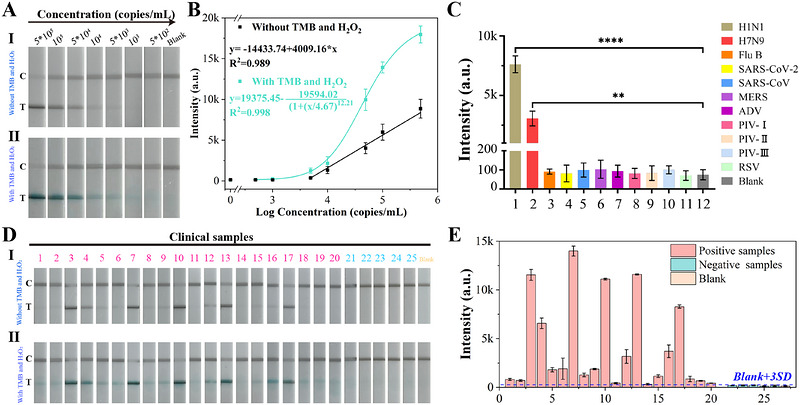
(A) Photographs of 3D magnetic multi‐metallic nanozymes‐based LFIA strips for inactive Flu A virus spiked in throat swab samples before (I) and after (II) catalytic reaction. (B) Calibration curves for inactive Flu A virus according to the detection results in (A). (C) Specific experiment by the 3D magnetic multi‐metallic nanozymes‐based LFIA strips. Photographs (D) and corresponding gray values (E) of clinical throat swab samples from 20 patients infected with Flu A and five negative samples.

To further evaluate the clinical applicability of this method, we detected a series of different concentrations of inactive Flu A virus solutions spiked in the throat swab samples via the 3D magnetic multi‐metallic nanozymes‐based LFIA strips. As shown in Figure 6A, the visual sensitivity for inactive Flu A (H1N1) virus before catalytic reaction was 5 × 10^3^ copies/mL. The corresponding gray values on the test lines before and after the catalytic reaction were used to plot the calibration curve (Figure 6B). By calculation, the LOD for inactive Flu A virus before and after catalytic reaction via the 3D magnetic multi‐metallic nanozymes‐based LFIA were 10^3.64^ and 140 copies/mL, respectively, a 30 times enhancement. Furthermore, several respiratory viruses, including H1N1, H7N9, Flu B, SARS‐CoV‐2, SARS‐CoV, MERS, ADV, PIV‐I∼III, and RSV were selected to verify the specificity of the proposed method. The photograph of related strips in Figure S19 showed invisible test lines for high concentrations of nontargeted respiratory viruses, except for low concentrations of the subtypes of Flu A (H1N1 and H7N9). Additionally, the gray values on the test lines of corresponding strips show that there is a significant difference between the subtypes of Flu A and the blank control, suggesting the excellent specificity of this method (Figure 6C).

To evaluate the precision and accuracy of our proposed method, we added 5, 0.5, and 0.05 ng/mL of Flu A nucleoprotein into the throat swab samples. The results shown in Table S3 suggested that the average recoveries ranged from 86%–119% and the RSD values were calculated to be 4.3%, 3.6%, and 6.5% for 5, 0.5, and 0.05 ng/mL of Flu A nucleoprotein, respectively. Moreover, 20 clinical positive throat swab samples collected from the Capital Institute of Pediatrics who infected with Flu A viruses, and five negative throat swab samples were also detected by the 3D magnetic MoS_2_@Fe_3_O_4_/Ag@AP‐based LFIA strips. The obtained samples were diluted 10 times with lysis buffer and added to the as‐prepared strips. The photographs and gray values of the corresponding strips are shown in Figure 6D,E. The *C*_t_ values of the 20 clinical positive throat swab samples were tested by qRT‐PCR, as shown in Table S4. However, only half of the positive samples can be detected by the commercial colloidal gold immunochromatographic strips at the same dilution times (Figure S20). In addition, the receiver operating characteristic (ROC) curve of the corresponding 20 positive and five negative samples was plotted, as shown in Figure S21, revealing the excellent accurate diagnosis of Flu A with an area under the curve (AUC) of 100%. The results demonstrated that all positive samples can be detected by our proposed trifunctional LFIA strips, suggesting their great precision and accuracy.

## 4 Conclusions

In conclusion, we reported a trifunctional LFIA based on the 3D magnetic multi‐metallic nanozymes for the detection of Flu A in clinical throat swab samples. Specifically, two‐dimensional MoS_2_ nanosheets with excellent physicochemical properties and large surface area served as the substrate of 3D magnetic multi‐metallic nanozymes. Satellite‐like Fe_3_O_4_/Ag nanoparticles and POD‐like AP NFs were then adsorbed on the surface of MoS_2_ nanosheets by the PEI‐mediated layer‐by‐layer assembly method. The synthesized 3D magnetic MoS_2_@Fe_3_O_4_/Ag@AP were used as immune labels to specifically capture the targets in the complex samples, providing colorimetric signals and catalytic abilities. Therefore, the introduction of 3D magnetic MoS_2_@Fe_3_O_4_/Ag@AP integrated three functions into one LFIA strip, namely, magnetic separation, colorimetric analysis, and catalytic amplification. Notably, the metals (Au and Ag) included in the 3D MoS_2_@Fe_3_O_4_/Ag@AP nanozymes further strengthened their POD‐like activity due to LSPR and the interaction of Pt/Au and Pt/Ag. Taking advantage of magnetic separation and catalytic amplification, the reported trifunctional LFIA strips can specifically capture and detect Flu A nucleoproteins and inactive Flu A viruses in throat swab samples with the LOD of 0.8 pg/mL and 140 copies/mL, respectively, 125 times lower than three commercial colloidal gold‐based LFIA strips. This method can complete the whole test within 26 min, and exhibits excellent specificity and accuracy. Additionally, 20 clinical throat swab samples infected with Flu A can be detected by the reported LFIA strips, revealing the reliability of the method in clinical application. This rapid and equipment‐free trifunctional LFIA has great application prospects in POCT for early and accurate diagnosis of highly pathogenic viruses.

## Author Contributions

Rui Xiao and Yansong Sun conceived and designed the project. Zhenzhen Liu performed the experiments and analysis and wrote the manuscript. Pengyou Zhou, Xiaofei Jia, and Xiaoxian Liu provided technical input on this project. All authors read and approved the final manuscript.

## Funding

The authors have nothing to report.

## Conflicts of Interest

The authors declare no conflicts of interest.

## Supporting information




**Supporting File**: exp270171‐sup‐0001‐SuppMat.docx.

## Data Availability

The data that supports the findings of this study are available in the supplementary material of this article.
