# Supplementary material for: Three‐Dimensional Magnetic Multi‐Metallic Nanozymes With Colorimetric and Catalytic Amplification for Rapid and Highly‐Sensitive Detection of Influenza A
Source: Exploration (Beijing). 2026 May 20;6(3):20240425. doi: 10.1002/EXP.20240425 (PMC13317654; doi:10.1002/EXP.20240425)
Supplement: Supplementary file 1 — Supporting File: exp270171‐sup‐0001‐SuppMat.docx. [file EXP2-6-20240425-s001.docx]

**Supporting Information**

**Three-dimensional magnetic multi-metallic nanozymes with colorimetric and catalytic amplification for rapid and highly-sensitive detection of influenza A**

Zhenzhen Liu^1^, Pengyou Zhou^1^, Xiaofei Jia^1^, Xiaoxian Liu^1^, Yong Yang^2^, Yansong Sun^1,^ *, Rui Xiao^1,^ *

^1^ State Key Laboratory of Pathogen and Biosecurity, Academy of Military Medical Sciences, Beijing 100071, China

^2^ State Key Laboratory of High-Performance Ceramics and Superfine Microstructures, Shanghai Institute of Ceramics, Chinese Academy of Sciences, Shanghai 200050, China

^🞶^Correspondence:

Rui Xiao and Yansong Sun, State Key Laboratory of Pathogen and Biosecurity, Academy of Military Medical Sciences, Beijing 100071, China

Email: ruixiao203@163.com; sunys6443@126.com

**S1. Material and Instruments**

Ferric chloride (FeCl_2_·4H_2_O), polyethyleneimine (PEI), ascorbic acid (AA), 11-mercaptoundecanoic acid (MUA), 2-(N-morpholino) ethanesulfonic (MES), N-hydroxysuccinimide (NHS), N-(3-dimethylaminopropyl)-N′-ethylcarbodiimide hydrochloride (EDC), bovine serum albumin (BSA), and 3,3′,5,5′-tetramethylbenzidine (TMB) were bought from Sigma-Aldrich (USA). MoS_2_ nanosheets was purchased from XFNANO. Polyvinylpyrrolidone (PVP, 50kDa) were purchased from Shanghai Macklin Biochemical Technology Co., Ltd. (China). hydrogen peroxide solution (H_2_O_2_, 30 wt%), acetic acid (HAc), chloroauric acid tetrahydrate (HAuCl_4_·4H_2_O) and NaOH were purchased from Shanghai Chemical Reagent Co. (China). Dihydrogen hexachloroplatinate hexahydrate (H_2_PtCl_6_·6H_2_O) were purchased from Thermo scientific. The components of LFIA strip were supplied by Jieyi Biotechnology Co., Ltd. (Shanghai, China), except nitrocellulose membrane which was purchased from Sartorius (Spain). Goat anti-mouse IgG was purchased from Sangon Biotech Co., Ltd. (Shanghai, China). Flu A nucleoprotein and mouse monoclonal anti-Flu A antibodies were procured from Fapon Biotech Inc. (China).

Transmission electron microscope (TEM) images of MoS_2_ nanosheets, MoS_2_@Fe_3_O_4_/Ag and MoS_2_@Fe_3_O_4_/Ag@AP were characterized via a Tecnai G2 F20 microscope (Philips). The zeta potential values and dynamic light scattering (DLS) data of the products during the process of synthesizing MoS_2_@Fe_3_O_4_/Ag@AP were acquired by a nano ZS90 zeta analyser (Malvern, UK). The UV–vis spectra of the materials were detected by a Shimadzu 3600 spectrometer. Magnetization saturations of the synthesized products were measured by vibrating sample magnetometer (Lake Shore 8604, America). In addition, the XPS and XRD patterns were measured by X-ray photoelectron spectrometer (Thermo escalab 250XI, America) and X-ray diffractometer (Brucker D8 Advance, Germany), respectively. Electron spin resonance (ESR) spectra were acquired by a Bruker A300 spectrometer. The absorbance values of oxTMB at 652 nm were recorded by Multifunctional microplate reader (Spectra Max i3x, America).

**S2. Preparation of Fe_3_O_4_/Ag nanoparticles and Au@Pt nanoflowers**

In brief, 3.75 mM of FeCl_2_·4H_2_O were dissolved in a flask containing 150 mL of deionized water and 20 mM of PVP were then added into them. After heating to 90℃, 10 mL of 1 M NaOH aqueous solution were added under stirring. While the temperature of the mixture reached 90℃ again, 40 mL of solution containing 2.5 mM of AgNO_3_ and 5 mM of PVP were added and heated for another 1 h. The products were then washed several times with deionized water and ethanol successively to remove the impurities. Finally, the synthetic Fe_3_O_4_/Ag were dried at 60℃ and stored in seal for further use. Moreover, Fe_3_O_4_ nanoparticles were synthesized by the same method but without AgNO_3_ precursor.

Au@Pt nanoflowers (AP NFs) were prepared as follows. Firstly, Au NPs (15 nm) were synthesized by sodium citrate reduction method. Briefly, 100 mL of 0.97 mM HAuCl_4_ were added in the flask and heated to boiling. 3 mL of 1 wt% TSC were then added under stirring and continued to heat for 15 min. After cooling down to room temperature,15 nm Au NPs were prepared and taken as Au seeds. Subsequently, 10 mL of 100 mM AA aqueous solution and 15 mL of 1 wt% H_2_PtCl_6_ were subsequently added to Au seeds. The mixture was placed in seal for 1 h at room temperature to growth the Pt shell on the surface of the Au NPs (AP NFs). In addition, Pt nanoparticles were produced by the same method but did not use Au NPs as the seed.

**S3. Preparation of** **MoS_2_@Fe_3_O_4_/Ag@Pt and MoS_2_@Fe_3_O_4_@AP**

MoS_2_@Fe_3_O_4_/Ag@Pt and MoS_2_@Fe_3_O_4_@AP were prepared by PEI-mediated layer-by-layer assembly method, the same as MoS_2_@Fe_3_O_4_/Ag@AP. Notably, the AP NFs were replaced by Pt nanoparticles in the synthetic process of MoS_2_@Fe_3_O_4_/Ag@Pt. Similarly, MoS_2_@Fe_3_O_4_@AP was prepared by replacing Fe_3_O_4_/Ag nanoparticles with Fe_3_O_4_ nanoparticles.

**S4. Assembly of the LFIA strip**

The LFIA strip consists of four parts: the PVC plate, the absorbent pad, the nitrocellulose membrane (CN140) and the sample pad. Notably, two lines (control line and test line) were set on the nitrocellulose membrane. Goat anti-mouse IgG (1 mg/mL) and anti-Flu A antibodies (1 mg/mL) were sprayed on control line and test line by the Biodot XYZ3050 plotter at a jetting rate of 1 μL/cm, respectively. The nitrocellulose membrane was then dried at 37℃ for 2 h. Finally, the absorbent pad, the nitrocellulose membrane and the sample pad were sequentially assembled on the PVC plate at a 2 mm distance from each other, and cut into 3 mm width for future use.

**S5. Computational details of density functional theory**

All spin-polarized density functional theory (DFT) calculations were performed using the Vienna Ab initio Simulation Package (VASP). The generalized gradient approximation (GGA) with the Perdew-Burke-Ernzerhof (PBE) functional was employed, and the projected augmented wave (PAW) potentials were used to describe the ionic cores. The valence electron wave functions were expanded in a plane-wave basis set with a kinetic energy cutoff of 400 eV. Partial occupancies of the Kohn−Sham orbitals were smeared using the Gaussian method with a width of 0.05 eV. The self-consistent convergence criterion for electronic energy was set to 10^−6^ eV, and a geometric optimization was considered convergent when the force change was smaller than -0.05 eV/Å^2^. Dispersion interactions were accounted for using Grimme’s DFT-D3 correction. For surface model optimizations, a 2×2×1 gamma-centered k-point grid was used to sample the Brillouin zone.


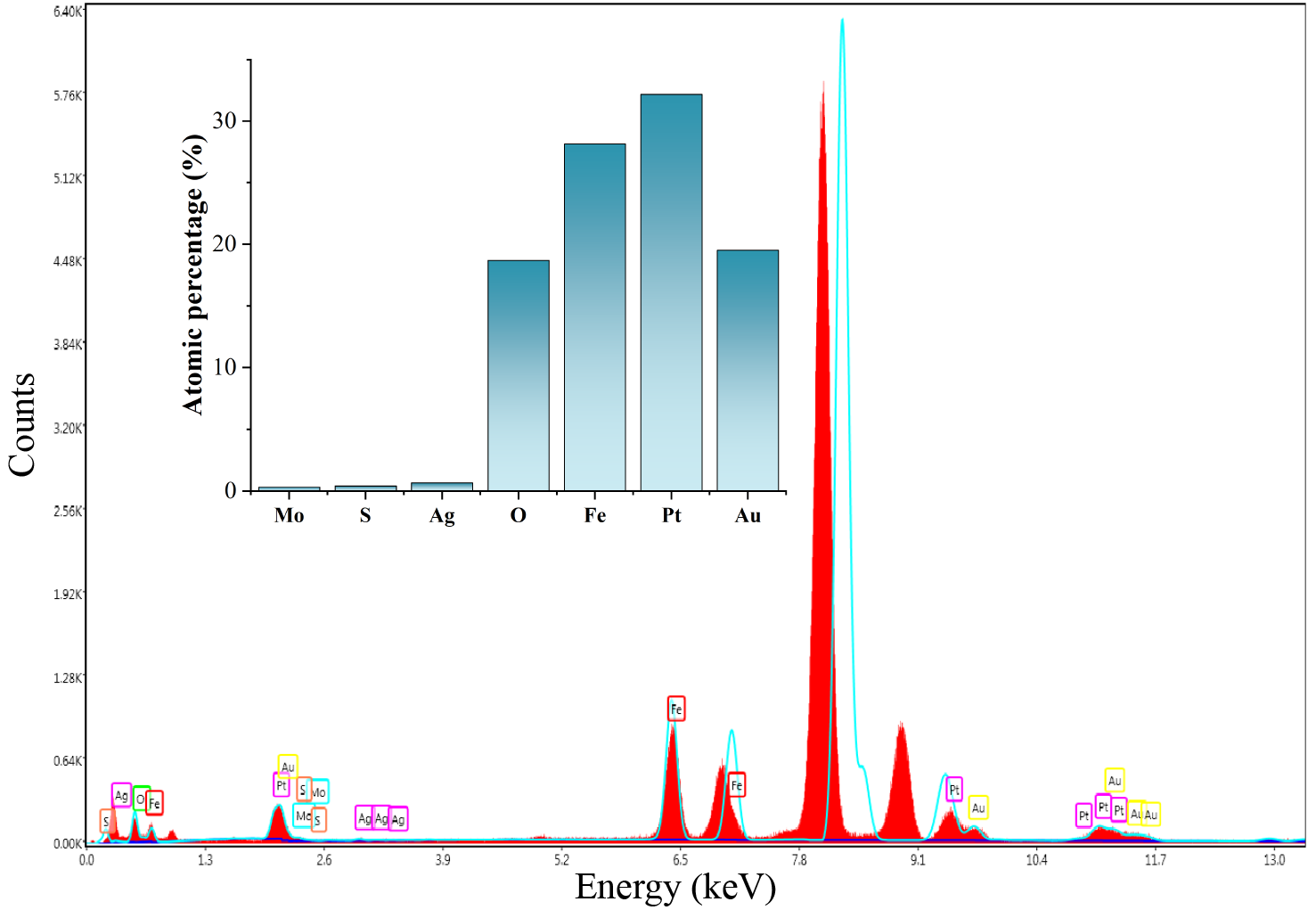


**FIGURE S1** EDS spectroscopy element distribution data of the MoS_2_@Fe_3_O_4_/Ag@AP.


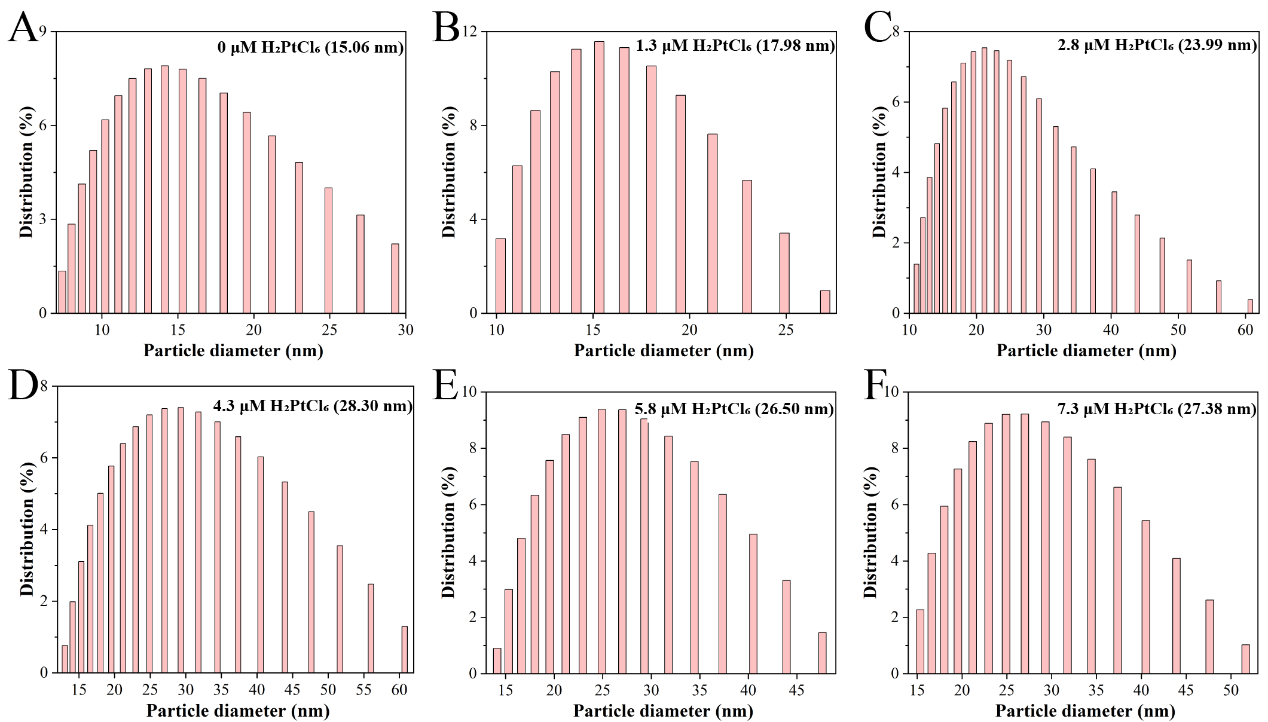


**Figure S2** The DLS distributions of AP NFs with different concentration of H_2_PtCl_6_: 1.3, 2.8, 4.3, 5.8 and 7.3 μM.


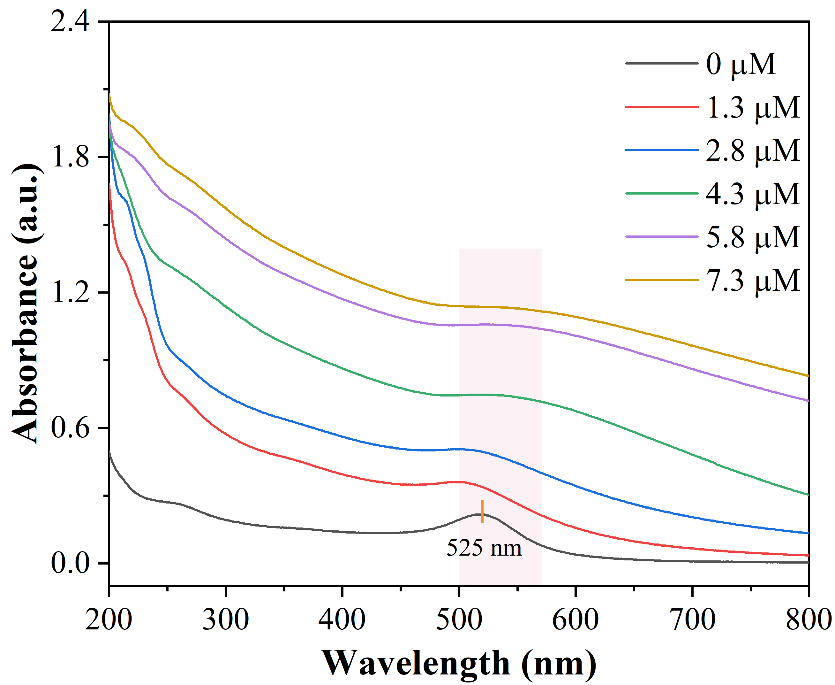


**Figure** **S3** The UV-vis spectra of AP NFs with different concentration of H_2_PtCl_6_ :0, 1.3, 2.8, 4.3, 5.8 and 7.3 μM.


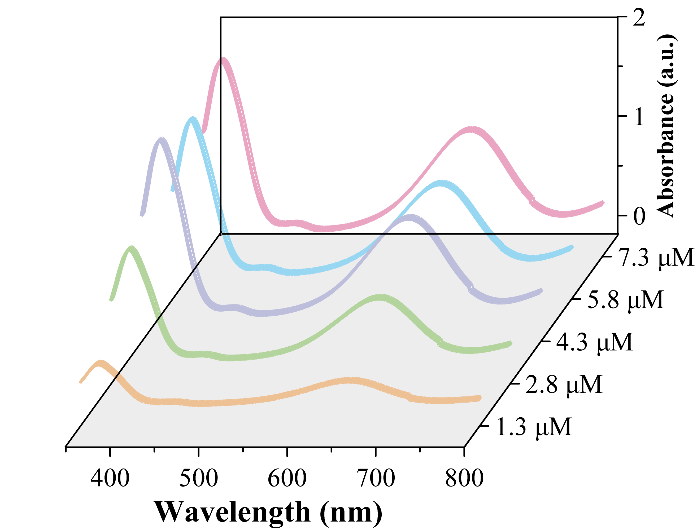


**Figure S4** UV-vis spectra of MoS_2_@Fe_3_O_4_/Ag@AP prepared by AP NFs with different concentrations of H_2_PtCl_6_: 1.3, 2.8, 4.3, 5.8 and 7.3 μM in acetate buffer solution containing H_2_O_2_ and TMB.


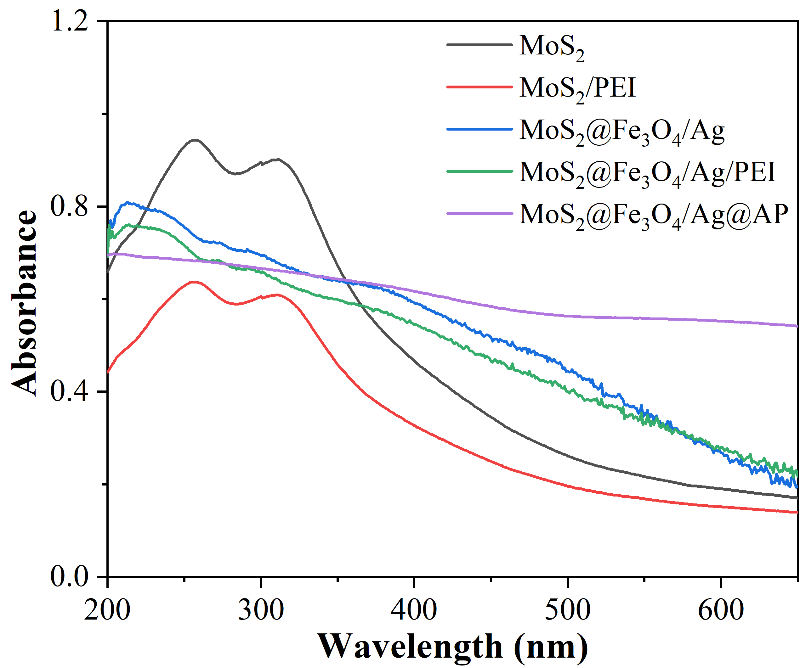


**FIGURE S5** UV-vis spectra of MoS_2_@Fe_3_O_4_/Ag@AP at different stages.


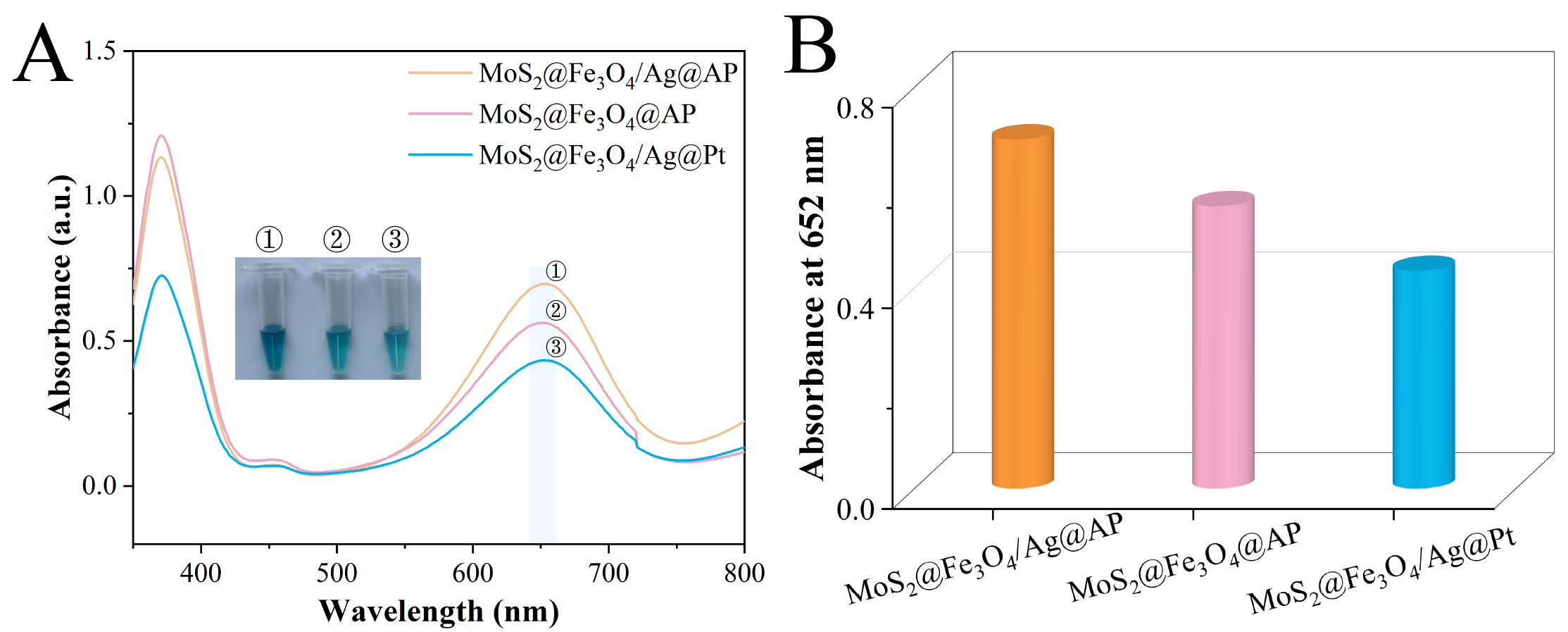


**Figure S6** UV-vis spectra (A) and the absorbance at 652 nm (B) of MoS_2_@Fe_3_O_4_/Ag@AP, MoS_2_@Fe_3_O_4_@AP and MoS_2_@Fe_3_O_4_/Ag@Pt in acetate buffer solution containing H_2_O_2_ and TMB. The inset photograph showed the corresponding solutions after catalytic reaction.


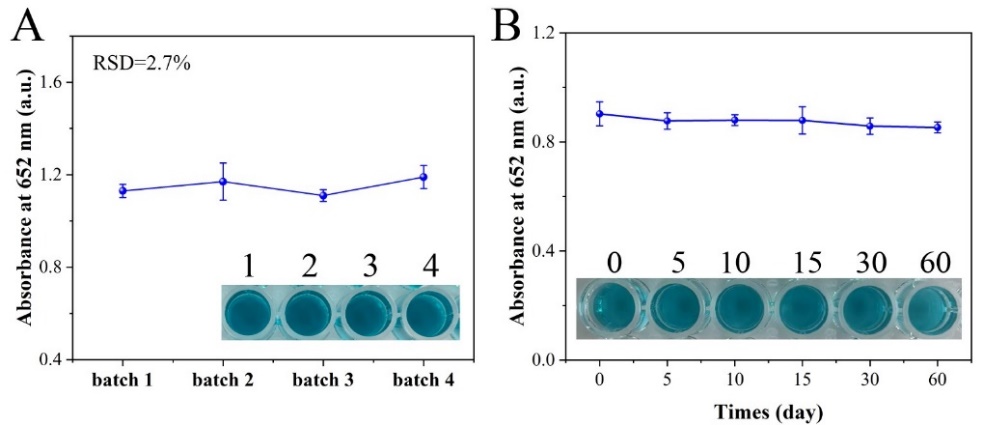


**FIGURE S7 (**A) Catalytic properties of four batches of MoS_2_@Fe_3_O_4_/Ag@AP in acetate buffer solution containing H_2_O_2_ and TMB. (**B)** Catalytic properties of MoS_2_@Fe_3_O_4_/Ag@AP after storage for 60 days. The inset shows the color of the corresponding solutions after catalytic reaction.


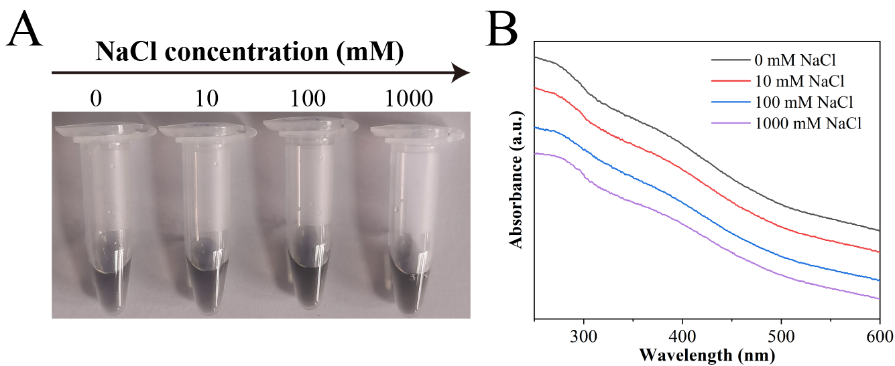


**Figure S8** Color changes (C) and corresponding UV–vis spectra (D) of MoS_2_@Fe_3_O_4_/Ag@AP at different salt concentrations (0–1000 mM NaCl).


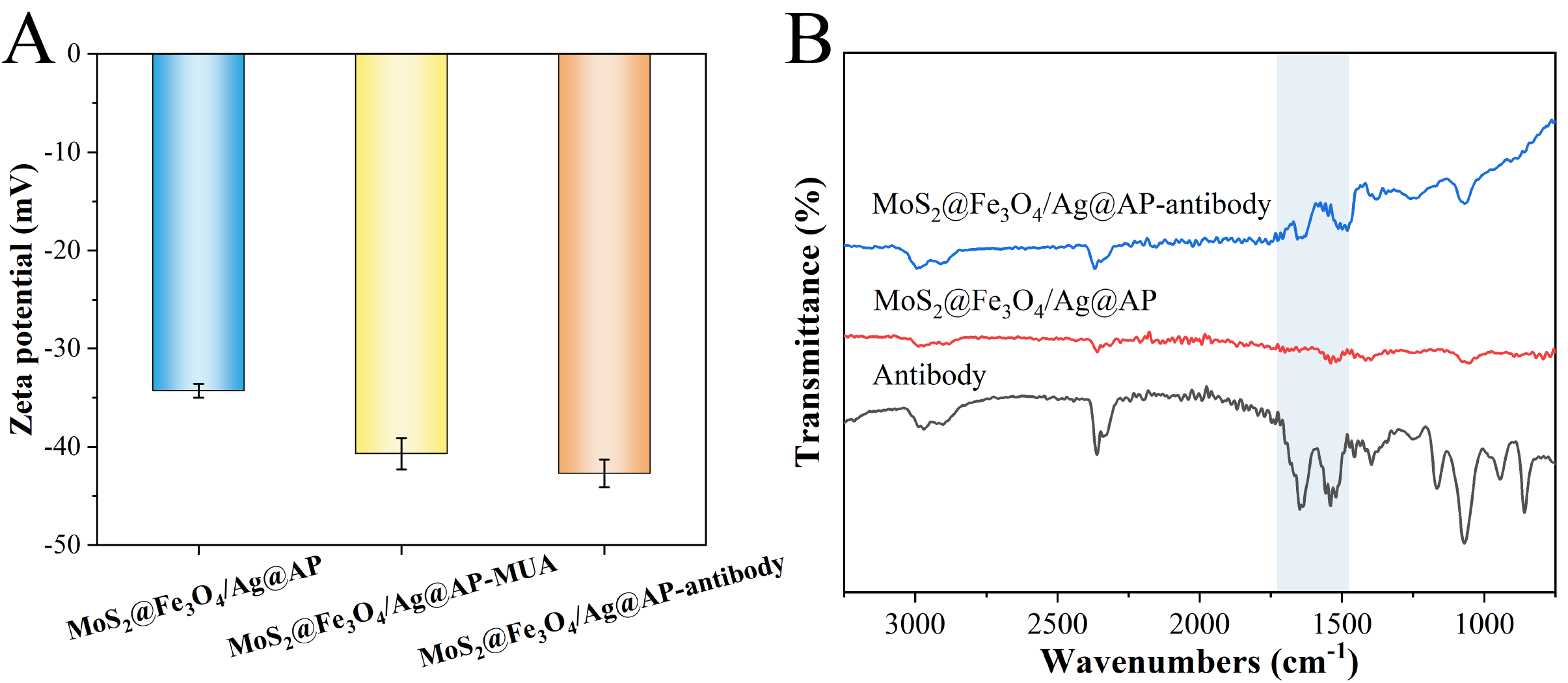


**Figure S9** (A) Zeta potentials MoS_2_@Fe_3_O_4_/Ag@AP, MoS_2_@Fe_3_O_4_/Ag@AP-MUA and MoS_2_@Fe_3_O_4_/Ag@AP-antibody. (B) FTIR spectra of antibody, MoS_2_@Fe_3_O_4_/Ag@AP and MoS_2_@Fe_3_O_4_/Ag@AP-antibody. The characteristic peaks at 1641 and 1530 cm^−1^ corresponded to protein amide bands I and II, respectively, appearing in immune labels reveal the success coupling of antibodies.


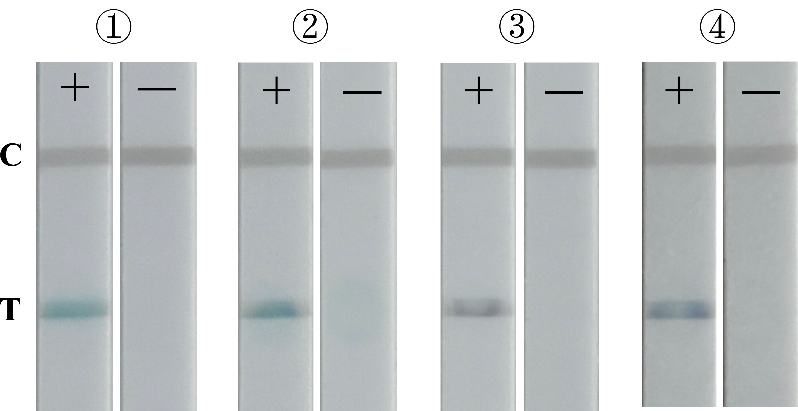


**Figure S10** Optimization of the chromogenic solution. TMB/H_2_O_2_ detection system, Precipitated one-component TMB substrate (ZOMANBIO)，TMB membrane substrate (Sigma), and TMB solution III (Biopanda).


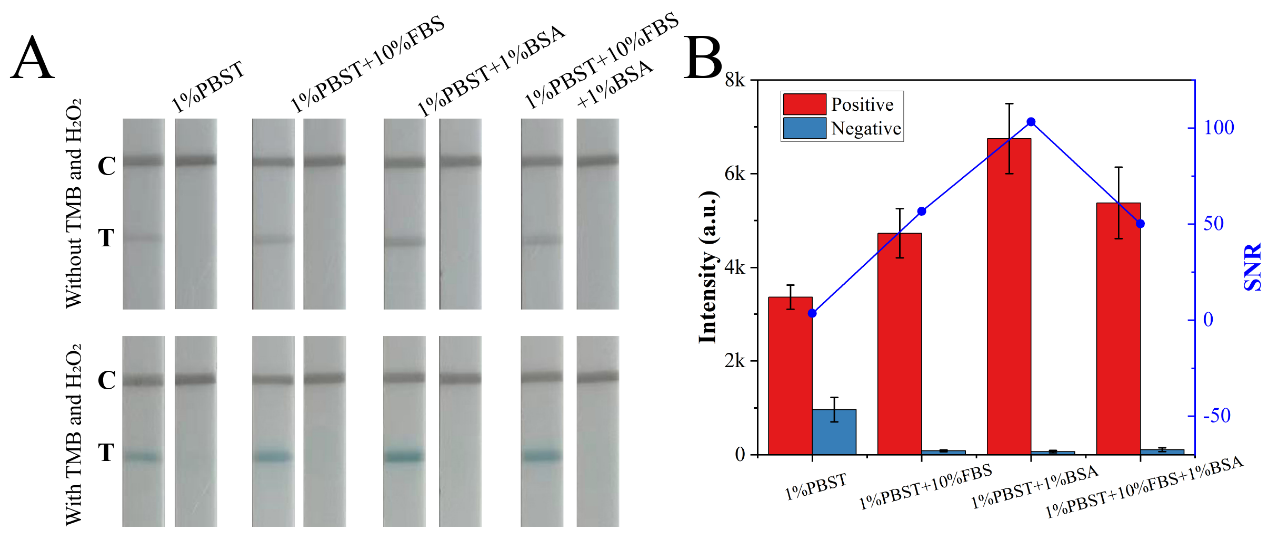


**Figure S11** Optimization of the loading buffer. Error bars were calculated from three independent experiments.


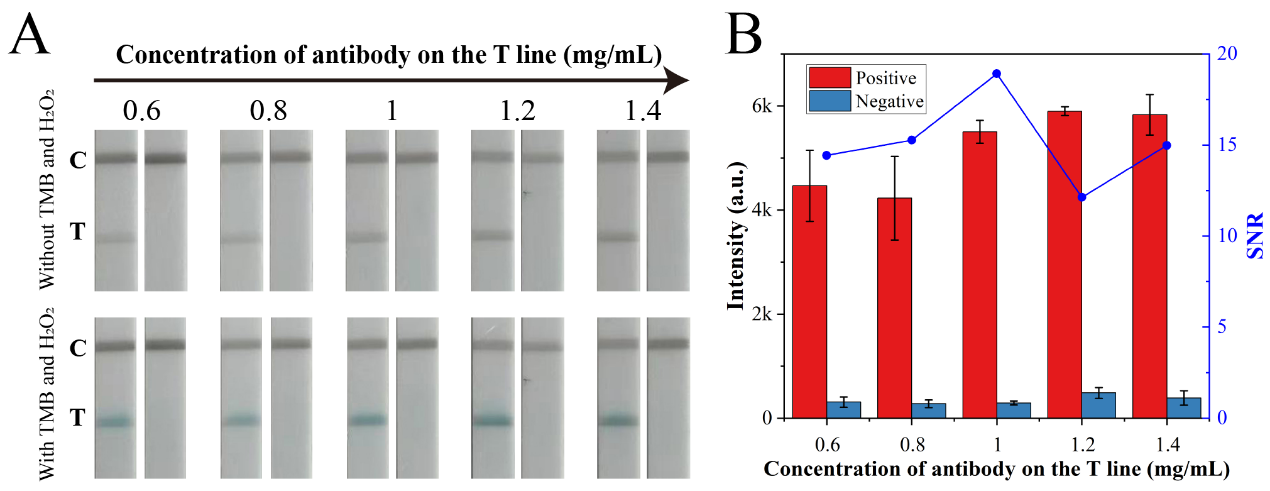


**Figure S12** Optimization of the concentration of antibody on the test line. Error bars were calculated from three independent experiments.


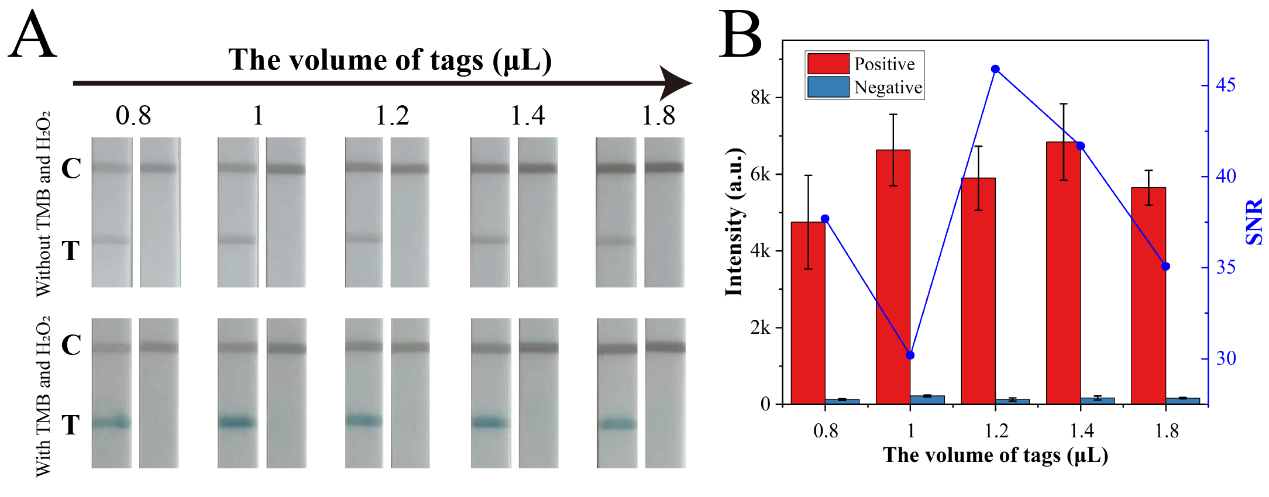


**Figure S13** Optimization of the addition of immune tags. Error bars were calculated from three independent experiments.


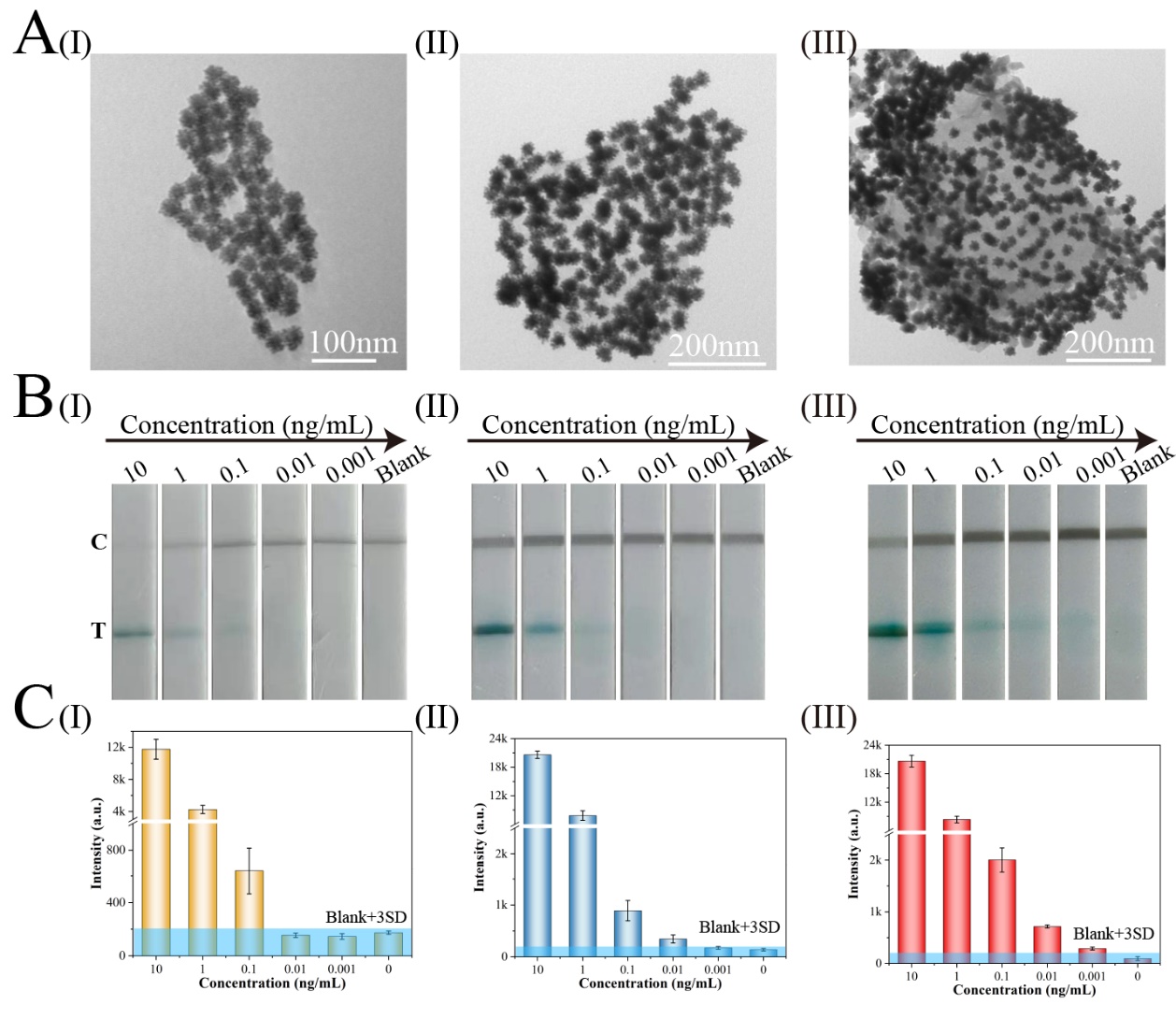


**Figure S14** (A) TEM images of AP NFs (i), MoS_2_@AP (ii) and MoS_2_@Fe_3_O_4_/Ag@AP (iii). (B) Photographs of AP NFs (i), MoS_2_@AP (ii) and MoS_2_@Fe_3_O_4_/Ag@AP (iii)-based lateral flow immunoassay strips with different concentration (10-0.001 ng/mL) of Flu A nucleoprotein. (C) Corresponding gray value of the test line for different concentration of Flu A nucleoprotein. Error bars indicated the standard deviations calculated from three separated experiments.


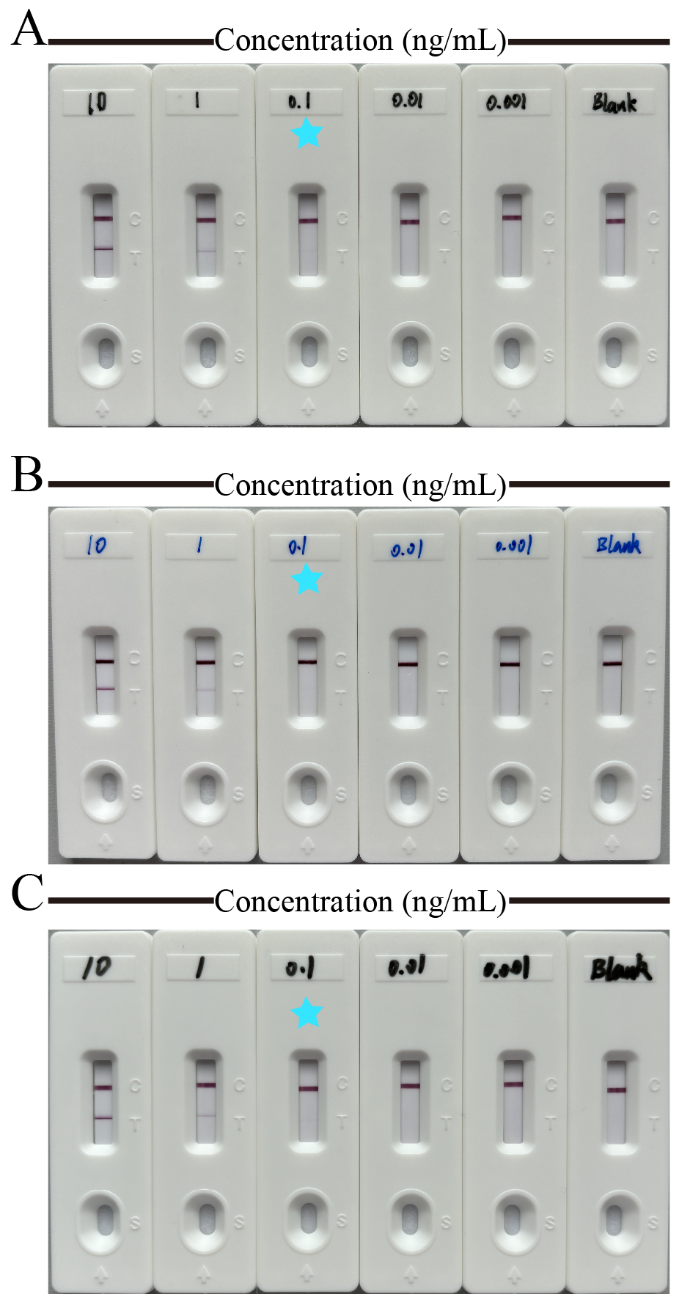


**Figure S15** Commercial colloidal gold immunochromatographic strips for Flu A nucleoprotein. From top to bottom, they were Biodragon (A), Easy Quarter (B) and Runbo Fude (C).


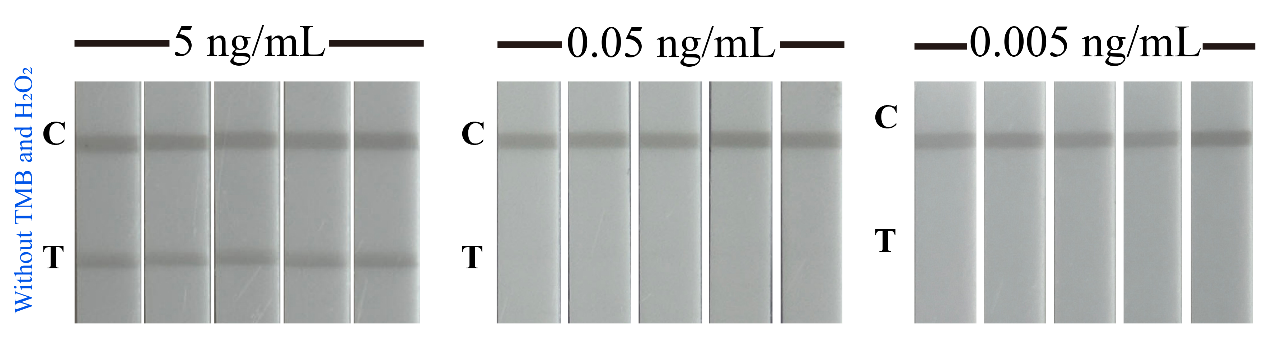


**FIGURE S16** Photographs of 3D magnetic multi-metallic nanozymes-based LFIA strips for repeated test before catalytic reaction with 5, 0.05 and 0.005 ng/mL of Flu A nucleoprotein.


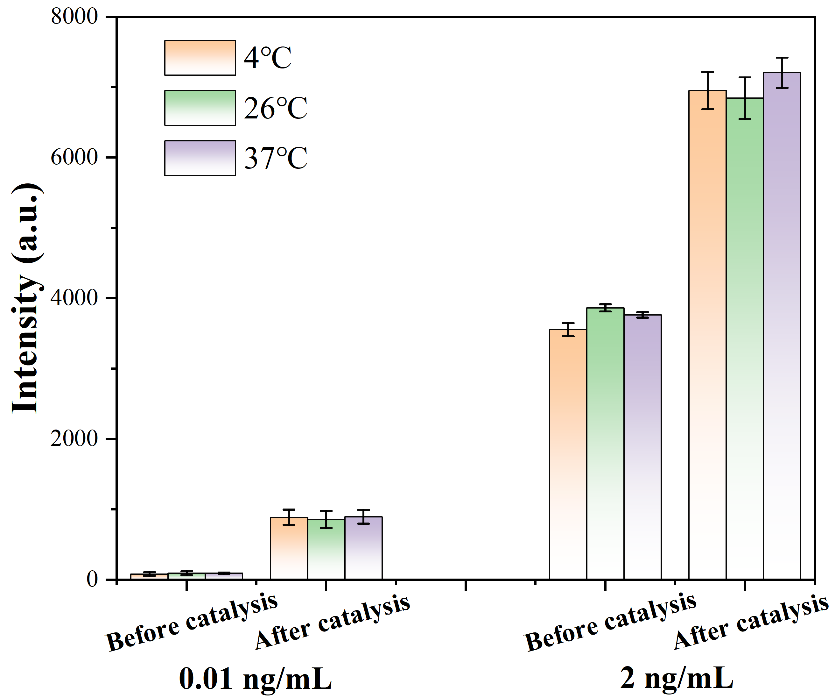


**FIGURE S17** Temperature stability of MoS_2_@Fe_3_O_4_/Ag@AP-based LFIA strips with 0.01 and 2 ng/mL of Flu A nucleoprotein before and after catalytic reaction.
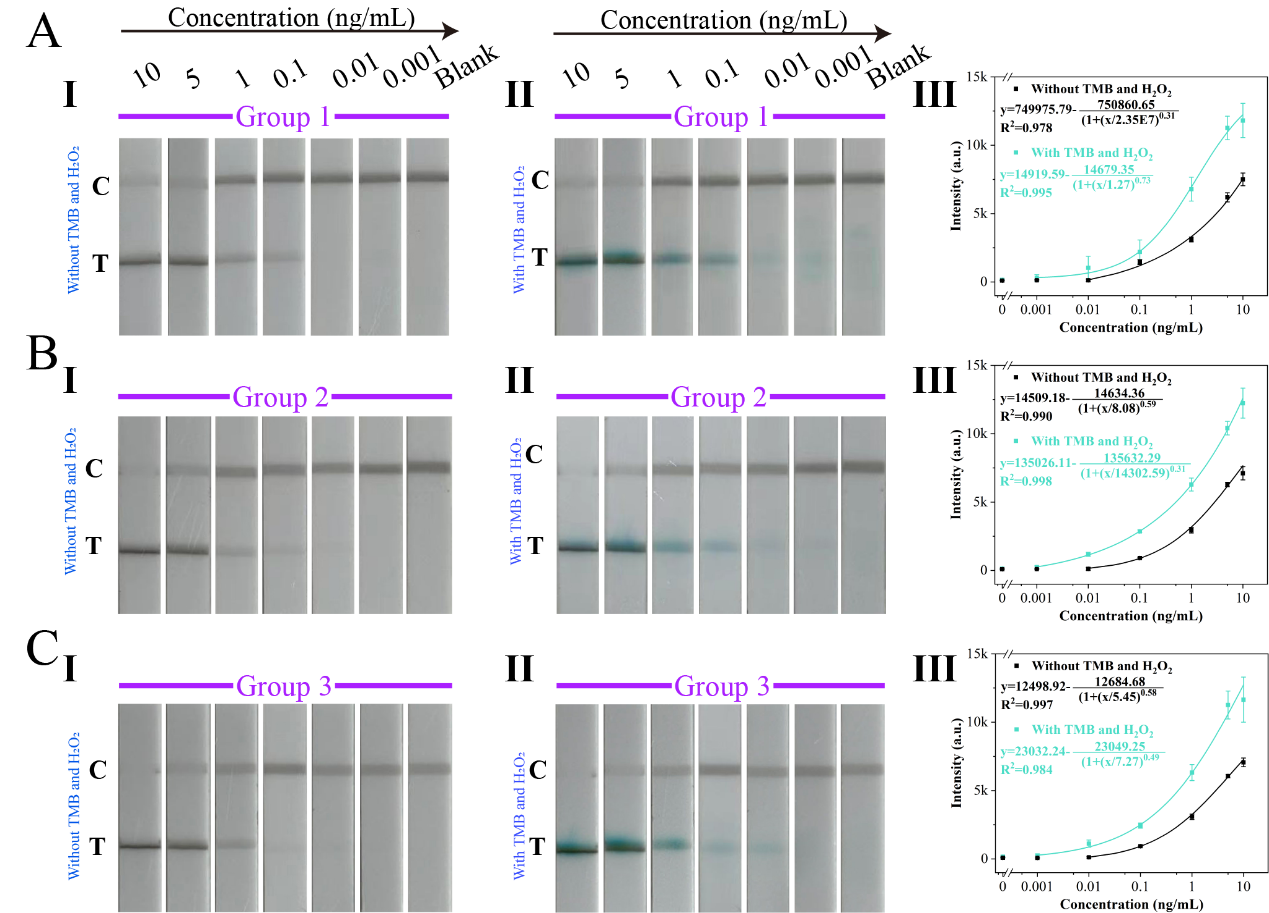


**FIGURE S18** Reproducibility of the 3D magnetic multi-metallic nanozyme-based LFIA via three batches of LFIA strips. Photographs of 3D magnetic multi-metallic nanozymes-based LFIA strips for Flu A nucleoprotein before (I) and after (II) catalytic reaction. III Calibration curve for Flu A nucleoprotein according to the gray values on the corresponding test lines.


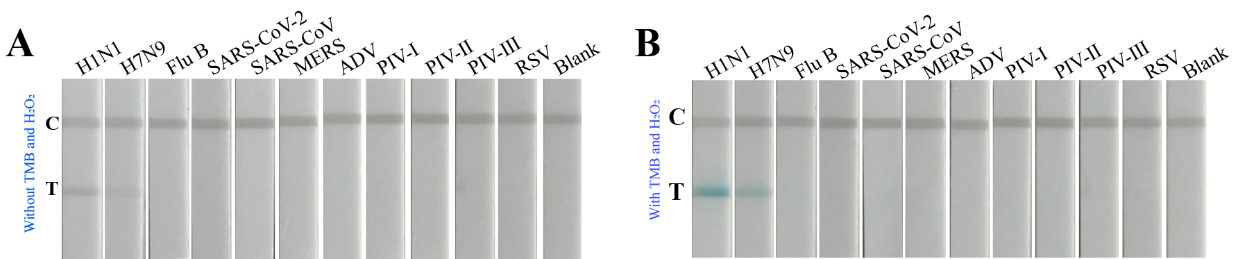


**FIGURE S19** Photographs of specific experiment before (A) and after (B) catalytic reaction via the 3D magnetic multi-metallic nanozyme-based LFIA.


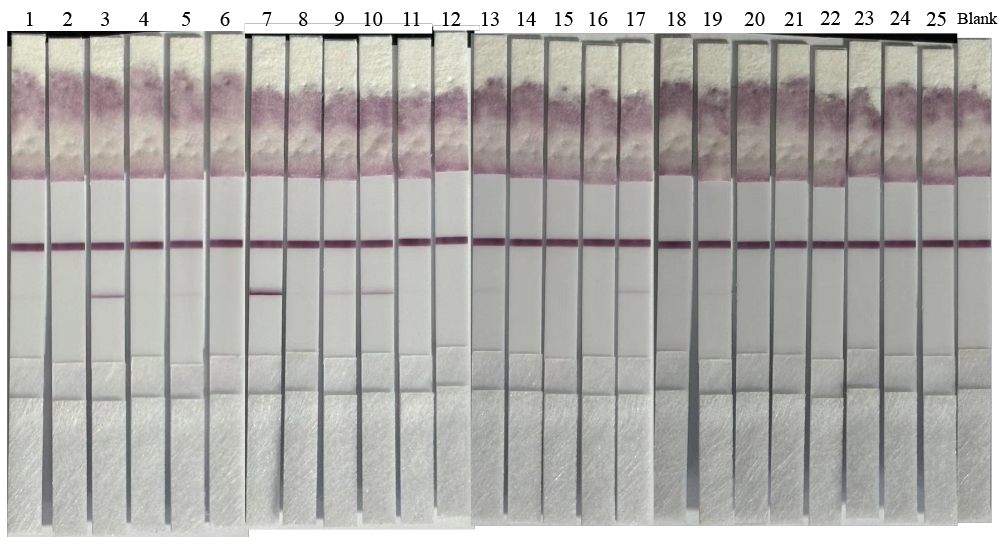


**Figure S20** Commercial colloidal gold immunochromatographic strips for 25 throat swab samples (20 positive and five negative samples).


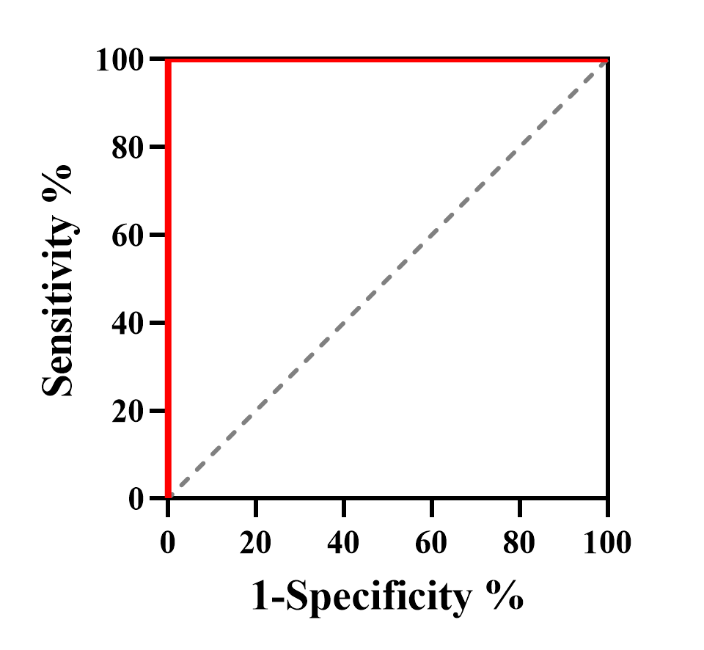


**Figure S21** ROC curve of the strips for 25 throat swab samples (20 positive and five negative samples) via the 3D magnetic multi-metallic nanozyme-based LFIA.

**Table S1**. Comparison of kinetic parameters of MoS_2_@Fe_3_O_4_/Ag@AP, HRP and other reported POD-like nanozymes.

| **Enzyme** | **Substrate** | ***K*m (mM)** | ***V*max (10^-6^ M/s)** | **Ref.** |  |
| --- | --- | --- | --- | --- | --- |
| HRP | H_2_O_2_  TMB | 0.63  0.82 | 0.0945  0.0543 | ^[1]^ |  |
| Fe_3_O_4_ | H_2_O_2_  TMB | 111  0.382 | 0.152  0.132 | ^[2]^ |  |
| Au@Pt | H_2_O_2_  TMB | 13.22  0.33 | 5.2  5.5 | ^[3]^ |  |
| Ag@Pt | | H_2_O_2_  TMB | 105.2  1.8 | 42.1  70.3 | ^[4]^ |
| Au@Pd@Pt NZs | H_2_O_2_  TMB | 4.59  0.065 | 0.19  0.25 | ^[5]^ |  |
| Fe_3_O_4_@MoS_2_-Au | H_2_O_2_  TMB | -  0.2 | -  0.079 | ^[6]^ |  |
| Fe_3_O_4_@MoS_2_@Pt | H_2_O_2_  TMB | 23.04  0.86 | 0.128  0.178 | ^[7]^ |  |
| **MoS_2_@Fe_3_O_4_/Ag@AP** | **H_2_O_2_**  **TMB** | **4.48**  **1.11** | **2.25**  **2.04** | **This work** |  |

**Table S2**. LODs for Flu A nucleoprotein before and after catalytic reaction via three batches of 3D magnetic multi-metallic nanozymes-based LFIA strips.

| **Batches** | LOD_Before_ (ng/mL) | LOD_After_ (ng/mL) |
| --- | --- | --- |
| **Group 1** | 0.011 | 0.0012 |
| **Group 2** | 0.010 | 0.009 |
| **Group 3** | 0.012 | 0.001 |
| **RSD (%)** | 9.09 | 14.78 |

**Table S3**. the recoveries of Flu A nucleoprotein in throat swab samples detected by the 3D magnetic multi-metals nanozymes-based LFIA.

| **Spiked (ng/mL)** | **Found (ng/mL)** | **Recovery (%)** | **RSD (%，n=3)** |
| --- | --- | --- | --- |
| 5 | 5.94 | 119 | 4.30 |
| 0.5 | 0.52 | 104 | 3.60 |
| 0.05 | 0.043 | 86 | 6.50 |

**Table S4**. The qRT-PCR results of 20 clinical positive throat swab samples.

| **Sample number** | **Viral**  **Ct** | **Sample number** | **Viral**  **Ct** | **Sample number** | **Viral**  **Ct** |
| --- | --- | --- | --- | --- | --- |
| 1 | 37.27 | 8 | 36.43 | 15 | 31.24 |
| 2 | 36.06 | 9 | 36.82 | 16 | 36.46 |
| 3 | 33.67 | 10 | 33.50 | 17 | 38.1 |
| 4 | 35.22 | 11 | 38.61 | 18 | 38.15 |
| 5 | 36.09 | 12 | 34.29 | 19 | 37.12 |
| 6 | 37.35 | 13 | 37.35 | 20 | 38.63 |
| 7 | 34.29 | 14 | 37.9 |  |  |

**S5. Ethical approval statement**

The clinical throat swab samples were approved by the Ethics Committee of Capital Institute of Pediatrics (approval number: SHERLL-2024-033). Written informed consent was obtained from all individual patients who provided the clinical throat swab samples used in this study. The samples were anonymized prior to analysis.

**REFERENCES**

[1] L. Gao, J. Zhuang, L. Nie, J. Zhang, Y. Zhang, N. Gu, T. Wang, J. Feng, D. Yang, S. Perrett and X. Yan, *Nat Nanotechnol* **2007**, *2*, 577.

[2] B. Xu, S. Li, L. Zheng, Y. Liu, A. Han, J. Zhang, Z. Huang, H. Xie, K. Fan, L. Gao and H. Liu, *Advanced Materials* **2022**, *34*.

[3] C. Lu, L. Tang, F. Gao, Y. Li, J. Liu and J. Zheng, *Biosensors and Bioelectronics* **2021**, *187*, 113327.

[4] Z. Du, L. Zhu, P. Wang, X. Lan, S. Lin and W. Xu, *Small* **2023**, *19*, e2301048.

[5] Y. Sun, Z. Xie, F. Pei, W. Hu, S. Feng, Q. Hao, B. Liu, X. Mu, W. Lei and Z. Tong, *Analytical Methods* **2022**, *14*, 5091.

[6] W. Xu, J. Fei, W. Yang, Y. Zheng, Y. Dai, M. Sakran, J. Zhang, W. Zhu, J. Hong and X. Zhou, *Microchemical Journal* **2022**, *181*, 107825.

[7] M. Xu, S. Zhao, C. Lin, Y. Li, W. Zhang, Y. Peng, R. Xiao, Z. Huang and Y. Yang, *ACS Applied Materials & Interfaces* **2024**, *16*, 11172.
